# Munropins G–J: Four New Prieurianin-Type Limonoids from *Munronia pinnata* and Their Structural and Molecular Characterization

**DOI:** 10.3390/ijms27073331

**Published:** 2026-04-07

**Authors:** Xuerong Yang, Jianxing Li, Peiyuan Liu, Xiaojie Yan, Fenglai Lu, Yoshiki Kashiwada, Xiangqin Li, Naonobu Tanaka, Dianpeng Li

**Affiliations:** 1 School of Pharmacy, Guangxi Health Science College, Nanning 530023, China; yxrxl@sina.cn (X.Y.);; 2 Guangxi Key Laboratory of Plant Functional Phytochemicals and Sustainable Utilization, Guangxi Institute of Botany, Guangxi Zhuang Autonomous Region and Chinese Academy of Sciences, Guilin 541006, China; 3 Graduate School of Pharmaceutical Sciences, Tokushima University, Tokushima 770-8505, Japan; 4 Guangxi Key Laboratory of Plant Conservation and Restoration Ecology in Karst Terrain, Guangxi Institute of Botany, Guangxi Zhuang Autonomous Region and Chinese Academy of Sciences, Guilin 541006, China; 5 Guangxi Academy of Sciences, Nanning 530007, China

**Keywords:** *Munronia pinnata*, Meliaceae, limonoids, structural elucidation, DP4+ analysis, TDDFT-ECD

## Abstract

*Munronia pinnata* (Meliaceae), a medicinal plant used in Zhuang traditional medicine, is recognized as a rich source of structurally diverse limonoids. In our continuing investigation of bioactive constituents from Guangxi medicinal plants, four new prieurianin-type limonoids, munropins G–J (**1**–**4**), were isolated from their aerial parts. Their structures were determined through comprehensive spectroscopic analysis, including nuclear magnetic resonance and high-resolution mass spectrometry, and further supported by quantum chemical calculations for electronic circular dichroism and statistical probability analysis. Munropins G (**1**) and H (**2**) feature an unprecedented C-12 β-D-glucosylated α-methyl-2′-hydroxypentanoate side chain and a C-17 β-substituted furan ring, with **1** being the 7-O-acetyl derivative of **2**. Munropins I (**3**) and J (**4**) possess a formyl group at C-11, a 3-methyl-2-hydroxypentanoate ester at C-12, and a C-17 γ-hydroxy-α,β-unsaturated γ-lactone unit (21-hydroxy for 3, 23-hydroxy for **4**), each existing as an equilibrating mixture of C-21 epimers—a phenomenon observed for the first time within a prieurianin-type framework. The absolute configurations of **1** and **2** were established by quantum chemical electronic circular dichroism calculations, while those of **3** and **4** remain to be assigned. All compounds were evaluated for cytotoxicity against human lung (A549), liver (HepG2), breast (MCF-7), and colon (HCT116) cancer cell lines and for anti-inflammatory activity in lipopolysaccharide-induced RAW 264.7 murine macrophages, but none exhibited significant effects at a concentration of 80 μM. This study expands the chemical diversity of *Munronia* limonoids and provides new molecular scaffolds for future structure–activity relationship investigations and chemotaxonomic markers for the Meliaceae family.

## 1. Introduction

*Munronia pinnata* (Wall.) W. Theob (synonyms: *M. henryi* Harms, *M. pumila* Wight, and *M. sinica* Diels) (Meliaceae), commonly known as “Aituotuo” in Chinese traditional medicine, is a perennial subshrub primarily distributed in limestone regions of Guangxi, China, with additional occurrences in Hainan, Yunnan, Guizhou, Chongqing, and Guangdong provinces [1,2]. This species is also naturally distributed in Sri Lanka, India, Indonesia, Malaysia, Vietnam, and the Philippines, where it has been extensively utilized in traditional medical systems for centuries [3]. Ethnopharmacological studies document its use in treating tuberculosis, cough, stomach pain, malaria, rheumatoid arthritis, and traumatic injuries through decoctions of roots, leaves, stems, or whole plants [4,5].

Recent pharmacological investigations have provided scientific validation for these traditional uses, demonstrating that extracts and fractions of *M. pinnata* exhibit a broad spectrum of bioactivities. Notably, crude extracts and fractions of Munronia species have been systematically evaluated for various pharmacological effects. The ethanol extract of *M. henryi* was demonstrated to exhibit significant analgesic and anti-inflammatory activities in rodent models, as evidenced by suppression of acetic acid-induced writhing; prolongation of hot-plate latency; and inhibition of xylene-induced ear edema, carrageenan-induced paw edema, and acetic acid-induced capillary permeability [6]. Acute toxicity studies established an oral median lethal dose (LD_50_) value of 478.9 mg/kg in mice, indicating a favorable safety margin at the recommended dosage [6]. Furthermore, aqueous decoctions of *M. pinnata* have been reported to exert hypoglycemic effects in both normal and alloxan-induced diabetic rats, with efficacy comparable to the standard drug glibenclamide, thereby providing scientific validation for its traditional use in diabetes management [7].

In addition to these systemic effects, limonoid-enriched fractions and isolated constituents from Munronia species have demonstrated potent antiviral activity. Several limonoids from *M. henryi*, including munronin O, exhibited remarkable inhibitory effects against tobacco mosaic virus (TMV), with half maximal effective concentration (EC_50_) values significantly lower than that of the commercial antiviral agent ningnanmycin [8,9]. Mechanistic studies revealed that these compounds act as plant resistance inducers by enhancing defense-related enzyme activities, such as phenylalanine ammonia-lyase (PAL), peroxidase (POD), polyphenol oxidase (PPO), and superoxide dismutase (SOD), upregulating salicylic acid accumulation, and activating the expression of pathogenesis-related genes pathogenesis-related protein 1a (PR-1a) and pathogenesis-related protein 5 (PR-5) [9]. In antimicrobial assays, mulavanin D and a pregnane-type steroid isolated from *M. delavayi* extracts showed moderate activity against *Microsporum gypseum* and *Trichophyton rubrum*, with minimum inhibitory concentration (MIC) values of 25–50 μg/mL [10]. Moreover, serum pharmacological studies using *M. henryi*-containing serum demonstrated pronounced antiproliferative and pro-apoptotic effects against H22 hepatoma cells, mediated through upregulation of Bcl-2-associated X protein (Bax) and Caspase-3, downregulation of B-cell lymphoma 2 (Bcl-2), and an increased Bax/Bcl-2 ratio [11]. These findings collectively highlight the therapeutic potential of Munronia-derived extracts and their limonoid constituents in oncology, infectious disease, and metabolic disorders.

Previous phytochemical studies on *M. pinnata* have shown its diverse secondary metabolites, including limonoids, triterpenoids, steroids, and diterpenoids [12,13,14,15]. Among these, limonoids, highly oxygenated tetranortriterpenoids, have attracted significant attention due to their structural complexity and broad biological activities, such as anti-cancer, anti-bacterial, anti-malarial, antiviral, and insect antifeedant properties [9,10,16,17]. The documented pharmacological effects of *M. pinnata* extracts are largely attributed to these limonoid constituents, establishing a clear structure–activity relationship (SAR) that motivates the isolation and characterization of novel limonoids from this species.

**Scheme 1:**
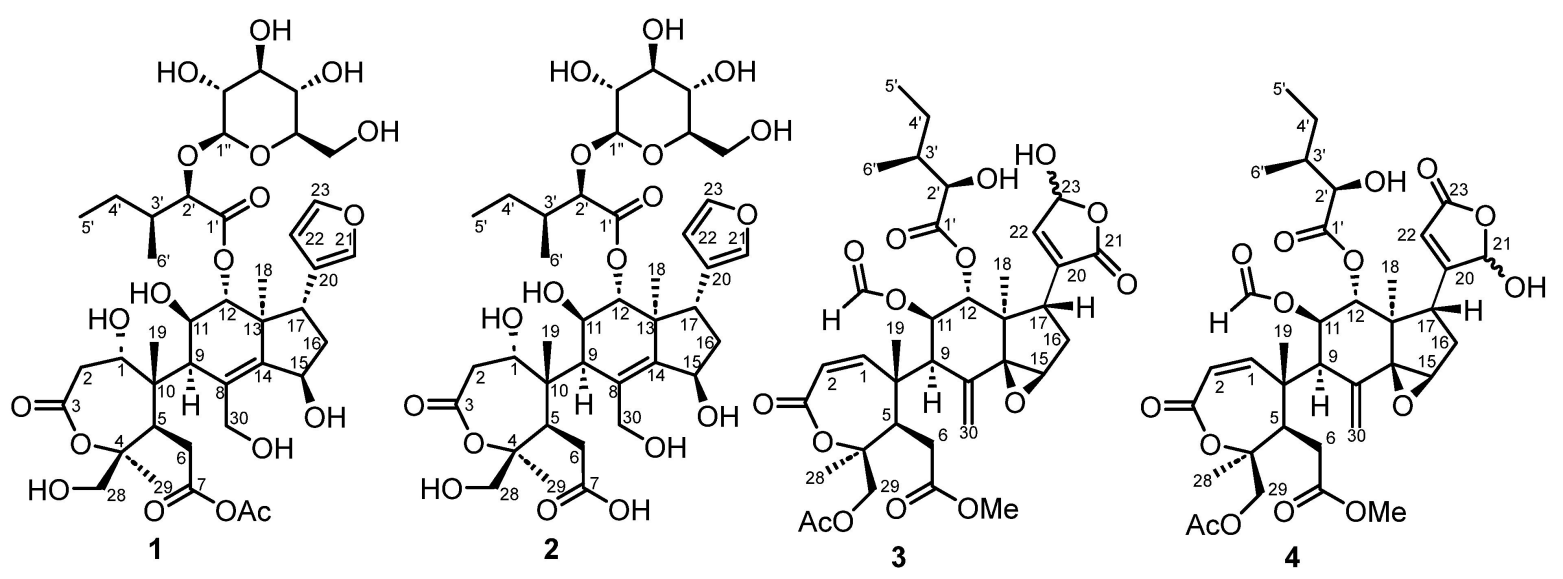
The structures of munropins G–J (**1**–**4**).

As part of our continuing investigation into the bioactive constituents of traditional herbal medicines from Guangxi, China, we have previously reported the isolation of five prieurianin-type limonoids (munropins A–E), one nimbolinin-type limonoid (munropin F), six new tirucallane-type triterpenoids, and a fatty acid glycoside from the aerial parts of *M. pinnata* [14,15,18]. These findings have underscored the remarkable chemical diversity and potential pharmacological relevance of this medicinal plant. Building upon this foundation, the present study further explores the limonoid constituents of *M. pinnata*, leading to the isolation and characterization of four new prieurianin-type limonoids, designated munropins G–J (**1**–**4**) (Scheme 1). Herein, we report the isolation, structural elucidation, and biological evaluation of these new compounds.

## 2. Results

### 2.1. Isolation and Structural Elucidation of New Limonoids

#### 2.1.1. Structure of Munropin G (**1**)

Phytochemical investigation of the 95% ethanol extract from the aerial parts of *Munronia pinnata* led to the isolation of four new limonoids, designated as munropins G–J (**1**–**4**) (Scheme 1). Their structures were elucidated by a comprehensive analysis of HRESIMS, 1D and 2D NMR spectra, and further confirmed by quantum chemical calculations.

Munropin G (**1**) was obtained as a white amorphous powder with optical activity {[a]_D_^20^ + 25.85 (*c* 0.1, MeOH)}. Its molecular formula was determined as C_40_H_58_O_19_ by HRESIMS (*m*/*z* 865.3483 [M + Na]^+^, calcd for 865.3465) (Figure S2), indicating 12 degrees of unsaturation. The IR spectrum showed absorption bands due to carbonyl (1722 and 1633 cm^−1^) and hydroxy (3425 cm^−1^) functionalities (Figure S9).

The ^1^H NMR spectrum (Figure S3) revealed the signals of a β-substituted furan ring; nine sp^3^ methines and six sp^3^ methylenes, of which five and two were oxygenated, respectively; and six methyls, including one acetyl, one triplet, one doublet, and three singlet methyls, as well as the resonances arising from a sugar moiety (Table 1). The DEPTQ NMR spectrum (Figure S4) displayed 40 carbon signals, including three carboxy carbonyl carbons, six olefinic carbons, one oxygenated tertiary carbon, and two quaternary carbons. These findings implied **1** to be an acylated limonoid glycoside.

**Figure 1:**
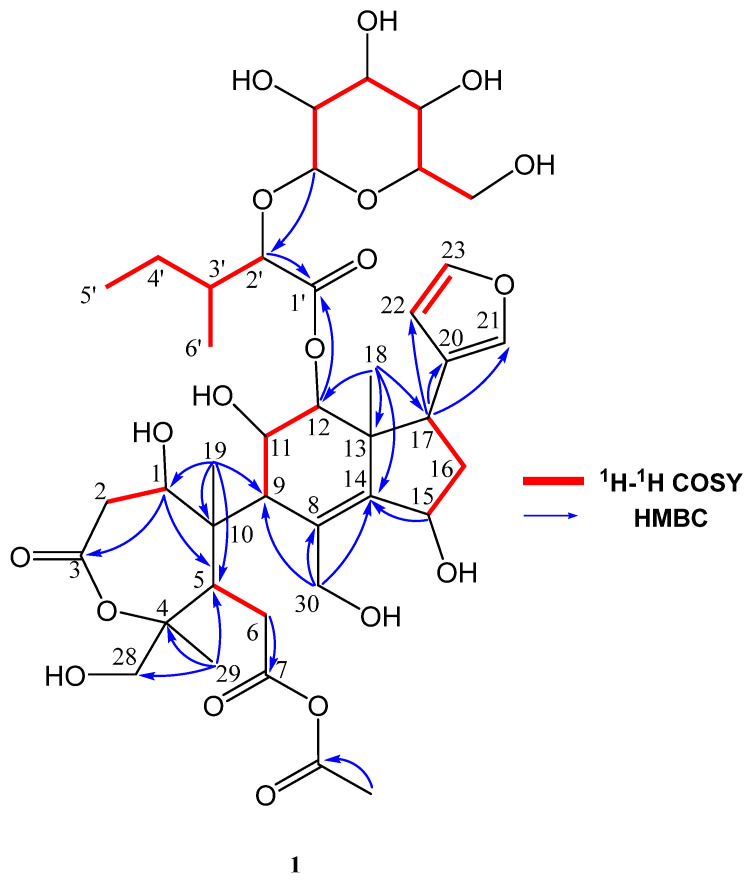
Key 2D-NMR correlations of munropin G (**1**).

The planar structure of **1** was established using 2D NMR correlations [^1^H-^1^H COSY (Figure S5), HSQC (Figure S6), HMBC (Figure S7)] (Figure 1). Key ^1^H-^1^H COSY correlations of H-9/H-11/H-12 and H-15/H_2_-16/H-17, along with HMBC correlations of H-15 with C-8/C-14, H_3_-18 with C-12/C-13/C-14/C-17, and H_2_-30 with C-8/C-9/C-14, indicated a hexahydroindene moiety (C-8, C-9, C-11–C-17) with a C-13 methyl and a C-8 oxygenated methine. HMBC correlations of H-12 with C-1′ and H-2′ with C-1′/C-3′/C-4′/C-6′, combined with ^1^H-^1^H COSY correlations of H-2′/H-3′/H_2_-4′/H_3_-5′/H_3_-6′, revealed an α-methyl-2′-hydroxypentanoic acid moiety (C-1′–C-6′) at C-12. A 1-hydroxy-4,10-dimethyl-4-oxymethylene-5-acetylcarbonylmethyl-ε-caprolactone ring was connected to the hexahydroindene moiety via C-9–C-10, supported by HMBC correlations of H-1 with C-3/C-5, H_3_-29 with C-4/C-5/C-28, and H_3_-19 with C-1/C-5/C-9/C-10. The β-substituted furan ring was linked to C-17 via C-20, as indicated by the HMBC correlation of H-17 with C-20.

**Figure 2:**
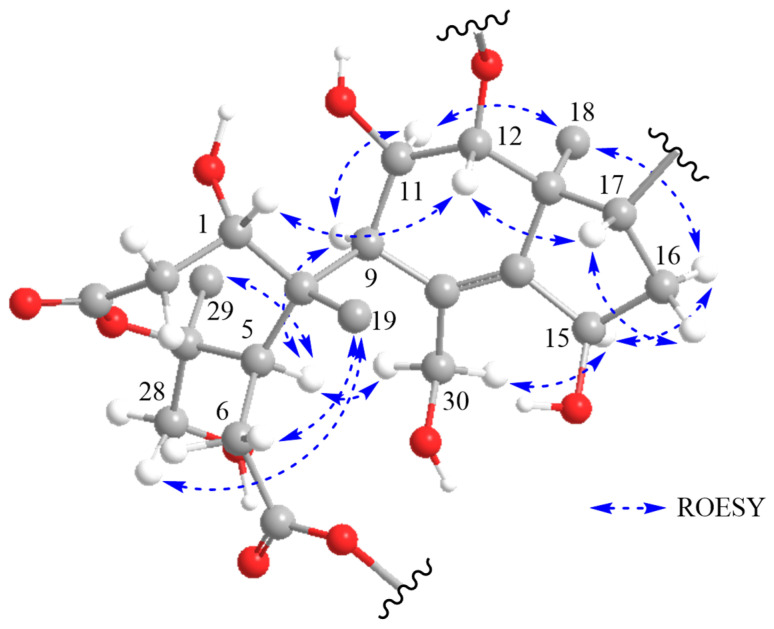
Key ROESY correlations and relative configuration for munropin G (**1**) (protons of methyl groups are omitted).

The sugar moiety of **1** was deduced as glucose on the basis of its proton coupling patterns and carbon chemical shifts. Treatment of the sugar moiety obtained by acid hydrolysis of **1** with *L*-cysteine methyl ester and *o*-tolylisothiocyanate, and high-performance liquid chromatography (HPLC) analysis of the reaction mixture gave a peak, whose retention time was identical to that of the derivative of an authentic sample of D-glucose prepared by an identical procedure [14]. A prieurianin-type skeleton of **1** was elucidated by 2D NMR analysis, including the ^1^H-^1^H COSY, HSQC, and HMBC analysis (Figure 2). This analysis also revealed the presence of hydroxy groups at C-1, C-11, C-12, C-15, C-28, and C-30, while the chemical shift of C-7 (dC 176.9) suggested it to be a carboxy carbonyl carbon. The presence of a 2-hydroxy-3-methylpentanoyl group at C-12 was indicated by ^1^H-^1^H COSY cross-peaks of H-2′/H-3′/H_2_-4′/H_3_-5′ and H-3′/H_3_-6′ as well as HMBC correlations for H-2′ and H-12 with C-1′. The connectivity of C-2′ and D-glycosyl group via the b-glycosidic linkage was confirmed by the coupling constant of the anomeric proton (H-1″, J = 7.9 Hz) and an HMBC correlation of H-1″ with C-2′. The absence of HMBC correlations between the acetoxy carbonyl carbon and any proton indicated that the acetyl group should be directly connected to the carbonyl group at position C-7. Therefore, the planar structure of munropin G (**1**) was assigned as shown in Figure 1.

The relative configuration of **1** was determined by ROESY spectroscopy (Figure S8). ROESY correlations of H-12/H-17, H-12/H-16, and H_2_-28/H_3_-19 indicated the cofacial b-orientations of these protons, while correlations of H-11/H_3_-18, H-16/H_3_-18, H-15/H-16, and H-9/H-11 assigned the a-orientations of H-5, H-9, H-11, H-15, and H_3_-18. The b-orientations of H-12 and H-17 and the a-orientations of H-9, H-11, H-15, and Me-18, as well as the *trans*-junction of the octahydroindene ring, were deduced by ROESY cross-peaks of H-12/H-17, H-11/H_3_-18, H-16a/H_3_-18, H-15/H-16a, H-30b/H-15, H-30a/H-9, and H-9/H-11. The 5R* and 10R* configurations were deduced by ROESY correlations for H-5/H-9, H-5/H_3_-28, H-5/H-30a, H_3_-19/H_3_-29, and H-2/H-12 (Figure 2). Thus, the structure of **1** was assigned as shown in Scheme 1.

**Figure 3:**
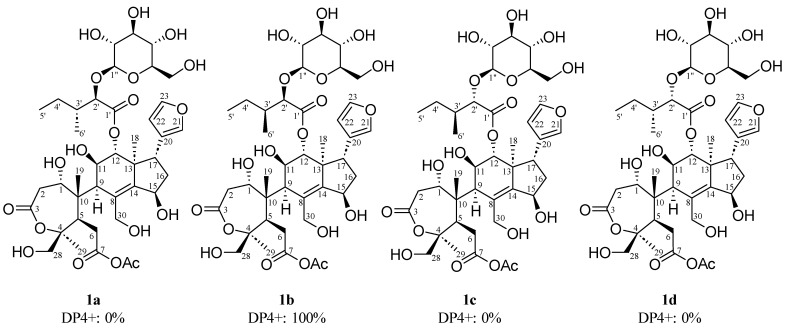
DP4+ analysis results for two possible configurations of compound **1**.

Determining the stereochemistry of flexible chain groups is one of the challenges in natural product chemistry research. For the confirmation of the relative configuration of the side chain in compound **1**, we employed NMR chemical shift calculation combined with DP4+ probability analysis [15] to determine the relative configurations at the 2′ and 3′ positions of compound **1**. Firstly, the Gauge Independent Atomic Orbitals (GIAO) method was used to calculate the theoretical chemical shifts of all carbon and hydrogen atoms for the four possible configurations of compound **1**, namely **1a** (2′R*, 3′R*), **1b** (2′R*, 3′S*), **1c** (2′S*, 3′S*), and **1d** (2′S*, 3′R*), at the mPW1PW91/6-311+G** (PCM, methanol) basis set level, followed by DP4+ probability analysis. The results showed that the probability of the **1c** configuration reached 100% (Figure 3). Therefore, the relative configurations at C-2′ and C-3′ of compound **1** were determined to be 2′R* and 3′S*.

**Figure 4:**
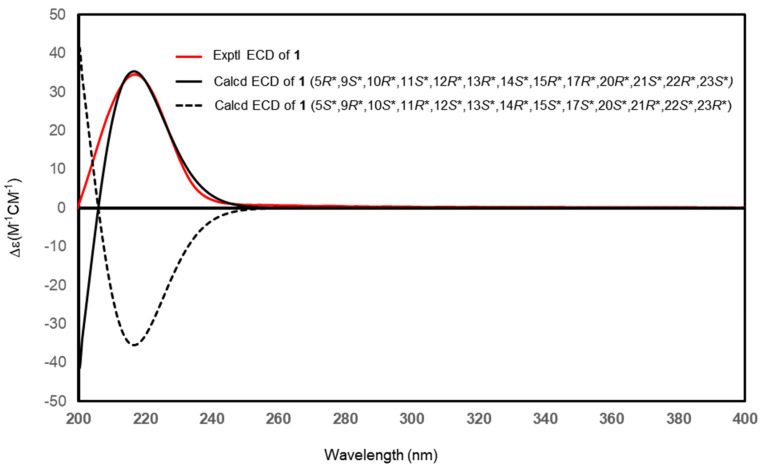
Experimental and calculated ECD spectra of munropin G (**1**).

The dependent density functional theory (TDDFT) calculation of a possible enantiomer-(1*S*, 4*S*, 5*R*, 9*S*, 10*R*, 11*S*, 12*R*, 13*R*, 15*S*, 17*R*, 2′*R*, 3′*S*) of **1** gave an electronic circular dichroism (ECD) spectrum (Figure 4), which corresponded to the experimental spectrum of **1**, suggesting the absolute configuration of munropin G (**1**) as shown in Scheme 1.

#### 2.1.2. Structure of Munropin H (**2**)

Munropin H (**2**) was obtained as a white amorphous powder with {[a]_D_^20^ + 25.9 (*c* 0.1, MeOH)}. Its molecular formula was C_38_H_56_O_18_ (HRESIMS: *m*/*z* 823.3601 [M + Na]^+^, calcd for 823.3511) (Figure S10), indicating 11 degrees of unsaturation. Comparison of 1D NMR data for **2** (Table 2, Figures S11 and S12) with those for **1** revealed the absence of an acetyl group signal in **2**, with a molecular weight difference corresponding to one acetyl group (C_2_H_2_O). Thus, **2** was identified as the deacetyl derivative of **1**. The relative configuration of **2** was deduced to be the same as **1** by the resemblance of their 1D NMR data (Table 1, Figures S13–S16). The ECD spectrum of munropin H (**2**) (Figure S17) was similar to that of **1**, suggesting that they had the same absolute configuration. Accordingly, the structure of munropin H (**2**) was elucidated as shown in Scheme 1. C_38_H_56_O_18_ (*m*/*z* 823.3601 [M + Na]^+^, calcd for 823.3511).

#### 2.1.3. Structures of Munropins I (**3**) and J (**4**)

Munropins I (**3**) and J (**4**) were obtained as optically active colorless amorphous solids {[α]_D_^21^ − 148.6 (*c* 0.1, MeOH) for **3**, [α]_D_^21^ − 127.2 (*c* 0.1, MeOH) for **4**}. The HRESIMS revealed they had the same molecular formula of C_36_H_46_O_15_ (*m*/*z* 741.2703 [M + Na]^+^, calcd for 741.2734; *m*/*z* 741.2724 [M + Na]^+^, calcd for 741.2734) (Figures S18 and S25) respectively. Analyses of the 1D and 2D NMR spectra (Figures S19–S24 and S26–S31) of **3** and **4** predicted they are similar to those of munropin C [18]. The observation of some duplicated signals in the 1D NMR spectra of **3** and **4** implied the existence of an equilibrium mixture of epimers (Table 2). The C-12 acetyl group in munropin C was replaced by a 3-methyl-2-hydroxy-pentanoate moiety as well as the C-11 acetyl group replaced by a formyl group in **3** and **4**, which was identified by the HMBC correlations of H-12 with C-1′ and H-2′ with C-1′, C-3′, C-4′, and C-6′ and H-11 with the formyl group, and COSY correlations of among H-2′/H-3′/H_2_-4′/H_3_-5′/H_3_-6′. Furthermore, the ^13^C NMR spectrum shows a 21-hydroxy-lactone group [δ_C_ 134.59 (C-20), δ_C_ 173.45 (C-21), δ_C_ 149.54 (C-22), δ_C_ 98.53; 98.33 (C-23)], which was located at C-17 in **3**, assigned by the HMBC correlations of H-17 (δ_H_) with C-20, C-21, and C-22 and H-22 with C-20, C-21, and C-23; moreover, a 23-hydroxy-lactone group located at C-17 in **4**, determined by the correlations of H-22 with C-20, C-21, and C-23, was assigned by the COSY correlation of H-22 with H-23 (Figure S1, Supporting Information).

Detailed analyses of the ROESY spectra of **3** and **4** indicated that their relative configurations were similar to munropin C, and the resemblance of their 1D NMR spectra also supported this assignment. The relative configuration of the 3-methyl-2-hydroxy-pentanoate moiety of **3** and **4** was deduced to be the same as **1** by the resemblance of their 1D NMR data (Table 2). The absolute configurations of **3** and **4** remain to be assigned.

### 2.2. Biological Activity Evaluation

The isolated compounds **1**–**4** were evaluated for their potential biological activities.

#### 2.2.1. Cytotoxicity Assay

At a concentration of 80 µM, none of the compounds (**1**–**4**) exhibited significant cytotoxic effects against a panel of four human cancer cell lines: human lung adenocarcinoma cell line (A549), hepatocellular carcinoma cell line (HepG2), breast adenocarcinoma cell line (MCF-7), and colorectal carcinoma cell line (HCT116) (Table S1).

#### 2.2.2. Anti-Inflammatory Activity

In a lipopolysaccharide (LPS)-induced nitric oxide (NO) production assay using RAW 264.7 murine macrophage cells, compounds **1**–**4** showed no significant inhibitory activity at 80 µM. Furthermore, they displayed no obvious cytotoxicity towards the RAW 264.7 cells at this concentration.

## 3. Discussion

The present phytochemical investigation of *Munronia pinnata* yielded four new prieurianin-type limonoids, munropins G–J (**1**–**4**), further expanding the already notable chemical diversity of this genus. Beyond their structural characterization, a critical examination of their distinctive features offers valuable insights into the chemodiversity, biosynthetic plasticity, and chemotaxonomic significance of prieurianin-type limonoids within the Meliaceae family.

From a structural perspective, compounds **1**–**4** share the characteristic prieurianin skeleton but exhibit notable variations at the C-17 side chain—a common site of structural diversification in this class. Munropins G (**1**) and H (**2**) are distinguished by a *β*-substituted furan ring at C-17 and a *β*-D-glucosylated α-methyl-2′-hydroxypentanoate moiety at C-12, with 1 being the acetylated derivative of 2. In contrast, munropins I (**3**) and J (**4**) feature a γ-hydroxy-α,β-unsaturated γ-lactone at C-17. The co-occurrence of both furan-ring-bearing (**1** and **2**) and γ-lactone-bearing (**3** and **4**) C-17 side chains within the same species is noteworthy. This structural dichotomy suggests the presence of divergent late-stage biosynthetic pathways in *M. pinnata*, likely involving oxidative modifications of a common furan precursor. The C-17 furan ring in **1** and **2** is typical of many Meliaceae limonoids, whereas the γ-hydroxy-α,β-unsaturated γ-lactone in **3** and **4** is less common and may represent a downstream oxidative product. The co-occurrence of both types within a single species provides a unique opportunity for future biosynthetic studies aimed at characterizing the enzymes responsible for this diversification. Another remarkable structural feature is the glycosylation at C-2′ in compounds **1** and **2**. While *O*-glycosylation is common in plant secondary metabolites, its occurrence on the acyl side chain of a limonoid is rare. This modification may affect the physicochemical properties of these compounds, such as solubility, stability, or subcellular localization, potentially influencing their ecological roles in the plant. Whether this glycosylation serves as a storage form, a transport vehicle, or a detoxification mechanism remains to be investigated. The rarity of this feature also makes it a potential chemotaxonomic marker for *M. pinnata* or related species.

The observation of equilibrating C-21 epimers in **3** and **4** represents the first report of such dynamic behavior within a prieurianin-type framework. This phenomenon, likely arising from hemiacetal formation at the lactone ring, adds a layer of complexity to the structural analysis of these compounds. Similar epimeric mixtures have been observed in other limonoid classes, such as the gedunin-type, but their occurrence in prieurianin-type limonoids is unprecedented. This finding underscores the structural flexibility of the C-17 side chain and should be carefully considered in future structural studies of lactone-bearing limonoids.

Comparison with known prieurianin-type limonoids reveals that munropins G (**1**) and H (**2**) are structurally related to munropin A [18], whereas munropins I (**3**) and J (**4**) share closer affinity with munropin C. Detailed structural comparison with munropin A highlights several distinctive modifications in **1**: the rearrangement of the Δ^8^,^30^ exocyclic olefin to a Δ^8^,^14^ endocyclic double bond, reductive opening of the C-14–C-15 epoxide to a C-15 hydroxy group, saturation of the Δ^1^,^2^ α,β-unsaturated-ε-lactone to a C-1 hydroxy functionality, and replacement of the C-4 methyl and C-7 methoxycarbonyl with an oxymethylene and an acetyl group, respectively. The C-11 and C-12 acetoxy groups in munropin A are replaced by hydroxy and α-methyl-2′-hydroxypentanoate functionalities, with the latter being further β-D-glucosylated at the 2′-hydroxy position—a structural feature unprecedented in munropin A. Most notably, the α,β-unsaturated γ-lactam moiety at C-17 in munropin A is replaced by a β-substituted furan ring in 1, representing a fundamentally different C-17 substitution pattern. These structural variations underscore the remarkable biosynthetic capacity of *M. pinnata* to generate diverse limonoid scaffolds through oxidation, reduction, glycosylation, and side-chain modification.

In contrast, munropins I (**3**) and J (**4**) are structurally akin to munropin C, with both compounds replacing the C-11 acetoxy group with a formyl functionality and the C-12 acetoxy moiety with a 3-methyl-2-hydroxypentanoate ester side chain. The C-17 side chains further differentiate the two compounds: munropin I (**3**) possesses a 21-hydroxy-γ-lactone ring, whereas munropin J (**4**) features a 23-hydroxy-γ-lactone unit. This positional isomerism at the lactone ring is rare within a single species and may reflect the action of regio-selective oxidative enzymes. The presence of both isomers in *M. pinnata* provides a valuable opportunity to study the enzymatic basis of lactone ring formation and functionalization.

From a chemotaxonomic perspective, the isolation of these four new limonoids reinforces the status of *Munronia* as a prolific producer of structurally diverse prieurianin-type limonoids. The co-occurrence of compounds with distinct C-17 side chains (furan vs. lactone) and varying oxidation patterns at C-11, C-12, and C-30 may serve as chemical fingerprints for *M. pinnata* and related species. Future chemotaxonomic studies could leverage these structural features to delineate species boundaries and phylogenetic relationships within the genus.

The biological evaluation revealed that compounds **1**–**4** did not exhibit significant cytotoxicity against the tested cancer cell lines or anti-inflammatory activity in LPS-induced macrophages at 80 μM. While these results are negative, they provide useful boundary information for future SAR studies. The absence of activity suggests that the specific structural features of these compounds—such as the glycosylated side chain in **1** and **2** and the hydroxylated γ-lactone in **3** and **4**—are not conducive to interaction with the biological targets involved in these particular assays. However, it remains possible that these compounds exhibit activity against other biological targets not assessed in this study, such as microbial pathogens, insect pests, or specific enzymes. The lack of cytotoxicity at 80 μM also positions these limonoids as potentially safe scaffolds for chemical modification aimed at introducing or enhancing bioactivity.

Future research directions arising from this work include: (1) investigation of the biosynthetic pathways responsible for the structural diversification observed in 1–4, particularly the furan-to-lactone conversion and the C-2′ glycosylation; (2) exploration of the ecological functions of these limonoids, such as insect antifeedant or antifungal activities, which are common in Meliaceae metabolites; (3) chemotaxonomic studies leveraging the structural features of **1**–**4** as markers for species identification and phylogenetic analysis within *Munronia*; and (4) targeted SAR studies using semisynthetic modification of these scaffolds to probe the structural requirements for specific bioactivities.

In conclusion, the discovery of munropins G–J enriches the chemical inventory of *Munronia pinnata* and highlights the remarkable structural plasticity of prieurianin-type limonoids. Their unique structural features—particularly the glycosylated side chain in **1** and **2**, the equilibrating lactone epimers in **3** and **4**, and the co-occurrence of furan and lactone C-17 side chains—provide valuable insights into the biosynthetic capacity and chemical diversity of this medicinal plant. These findings lay a foundation for future biosynthetic, ecological, and chemotaxonomic investigations and contribute to the broader understanding of limonoid diversity in the Meliaceae family.

## 4. Materials and Methods

### 4.1. Plant Material

The aerial parts of *Munronia pinnata* (Wall.) W.Theob. (Meliaceae) were collected in Jingxi County, Guangxi Zhuang Autonomous Region, China, in July 2021. The plant material was authenticated by Prof. Dianpeng Li (Guangxi Academy of Sciences). A voucher specimen (No. 21-GX-001) has been deposited at the herbarium of the Center for Natural Products Chemistry Studies, Guangxi Institute of Botany, Chinese Academy of Sciences. This species, known in traditional Zhuang medicine as “Aituotuo,” is widely distributed in the karst limestone regions of Guangxi and neighboring provinces. To date, no genomic information for *M. pinnata* has been reported in the public domain, warranting further investigation in future studies.

### 4.2. Instruments and Reagents

The following instruments were employed for experimental analyses: a Bruker Avance 500 MHz nuclear magnetic resonance (NMR) spectrometer for acquiring 1D and 2D NMR spectra (Bruker Corporation, Billerica, MA, USA); an Agilent 1260 high-performance liquid chromatography (HPLC) system (Agilent Technologies, Santa Clara, CA, USA) for preparative isolation and purification; a Shimadzu LCMS-IT-TOF mass spectrometer (Shimadzu Corporation, Kyoto, Japan) for determining high-resolution electrospray ionization mass spectrometry (HRESIMS) data; a Jasco P-1020 polarimeter (Jasco Corporation, Tokyo, Japan) for measuring optical rotations; and a Jasco J-810 circular dichroism (CD) spectrometer (Jasco Corporation, Tokyo, Japan) for recording electronic circular dichroism (ECD) spectra.

All chemical reagents used in the study, including methanol, ethanol, and acetonitrile, were of analytical grade or chromatographic grade to ensure the accuracy and reliability of experimental results. Chromatographic materials utilized for separation and purification procedures included silica gel (300–400 mesh) (Qingdao Haiyang Chemical Co., Ltd., Qingdao, China) for column chromatography, Sephadex LH-20 gel (Cytiva, Marlborough, MA, USA) for size-exclusion chromatography, and reversed-phase C18 (RP-C18) gel (Merck KGaA, Darmstadt, Germany) for reversed-phase column chromatography.

### 4.3. Extraction and Isolation

Dried aerial parts of *M. pinnata* (25 kg) were pulverized and extracted with 95% ethanol (150 L × 4) at room temperature. The concentrated crude extract (427 g) was suspended in water and partitioned successively with petroleum ether, ethyl acetate, and water to afford petroleum ether- (199 g), ethyl acetate- (199 g), and water-soluble (119 g) fractions. The water-soluble fraction (119 g) was subjected to HPD macroporous resin column chromatography eluting with ethanol–water (20:80 → 80:20, *v*/*v*) to yield Fr.1–Fr.3. Fr.2 (80% ethanol eluate, 18 g) was purified by Sephadex LH-20 (methanol) and RP-C18 column chromatography (methanol-water, 30:70 → 50:50, *v*/*v*), affording Fr.2.1–Fr.2.7. Fr.3 (40% ethanol eluate, 46 g) was separated by silica gel column chromatography (dichloromethane–methanol, 90:10 → 0:100, *v*/*v*) to give Fr.3.1–Fr.3.20. Compound **1** (38.0 mg) was isolated from Fr.2.2 by high-speed counter-current chromatography (HSCCC) using dichloromethane–methanol–water (2:2:1, *v*/*v*/*v*, aqueous stationary phase) followed by preparative HPLC. Compound **2** (13.6 mg) was obtained from Fr.3.4 using the same HSCCC method.

In a separate extraction, dried aerial parts (1.64 kg) were extracted with methanol (3 L × 3) to yield a crude extract (111.4 g), which was partitioned into an ethyl acetate-soluble fraction (34.2 g). This fraction was separated by silica gel column chromatography (n-hexane–ethyl acetate, 90:10 → 25:75, *v*/*v*) to afford Fr.1–Fr.12. Fr.11 was further purified by MCI gel CHP20P (methanol) to give Fr.11.1–Fr.11.5. Subsequent gel permeation chromatography (GPC) and octadecylsilyl (ODS) HPLC (30% aqueous acetonitrile, *v*/*v*) of Fr.11.2.5 and Fr.11.3.3 afforded munropin I (**3**, 2.5 mg) and munropin J (**4**, 2.3 mg), respectively.

#### 4.3.1. Munropin G (**1**)

Colorless solid; [α]_D_^20^ + 25.85 (*c* 0.1, MeOH); UV (MeOH) λ_max_ (log ε) 208 (4.5) nm; CD (MeOH) Δε (nm) + 38.2 (219); ^1^H and ^13^C NMR data (Table 1); HRESIMS *m*/*z* 865.3483 [M + Na]^+^ (calcd for C_40_H_58_O_19_Na, 865.3465).

#### 4.3.2. Munropin H (**2**)

Colorless solid; [α]_D_^20^ − 36.35 (*c* 0.1, MeOH); UV (MeOH) λ_max_ (log ε) 209 (4.3) nm; CD (MeOH) Δε (nm) + 13.1 (218); ^1^H and ^13^C NMR data (Table 1); HRESIMS *m*/*z* 823.3601 [M + Na]^+^ (calcd for C_38_H_56_O_18_Na, 823.3511).

#### 4.3.3. Munropin I (**3**)

Colorless solid; [α]_D_^21^ − 148.6 (*c* 0.1, MeOH); UV (MeOH) λ_max_ (log ε) 209 (4.6) nm; CD (MeOH) Δε (nm) + 10.3 (202) and +3.3 (252); ^1^H and ^13^C NMR data (Table 2); HRESIMS *m*/*z* 741.2703 [M + Na]^+^ (calcd for C_36_H_46_O_15_Na, 741.2734).

#### 4.3.4. Munropin J (**4**)

Colorless solid; [α]_D_^21^ − 127.2 (*c* 0.1, MeOH); UV (MeOH) λ_max_ (log ε) 210 (4.9) nm; CD (MeOH) Δε (nm) +12.7 (208) and +11.8 (248); ^1^H and ^13^C NMR data (Table 2); HRESIMS *m*/*z* 741.2724 [M + Na]^+^ (calcd for C_36_H_46_O_15_Na, 741.2734).

### 4.4. Conformational Search and NMR Calculations

Within an energy window of 0–20.93 kJ/mol, conformational random searches were performed using Spartan 14 V1.1.4 software under the MMFF94S force field. The results showed that each of the compounds **1a**, **1b**, **1c**, and **1d** had 10 low-energy conformations. To further verify the stability of these conformations, density functional theory (DFT) calculations were applied: the low-energy conformations were optimized using Gaussian 09 software at the B3LYP-D3(BJ)/6-31G(d)* level [19]. After excluding conformations with energies exceeding 20.93 kJ/mol, 7 stable conformations remained for both **1a** and **1b**. These conformations were confirmed to have no imaginary frequencies, ensuring they were true minima on the potential energy surface.

For subsequent NMR calculations of these stable conformations, the GIAO method implemented in the Gaussian 09 program was employed to calculate the carbon and hydrogen atomic chemical shielding values at the mPW1PW91/6-311+G** level with the polarizable continuum model (PCM) using methanol as the solvent. Meanwhile, the chemical shielding values of carbon and hydrogen atoms in the reference compound tetramethylsilane (TMS) were calculated following the identical procedure and level of theory. The theoretical chemical shifts of carbon and hydrogen atoms for each conformation were obtained by subtracting the shielding values of individual conformations from the average shielding value of TMS. Finally, the carbon and hydrogen chemical shifts of **1a** and **1b** were derived based on the Boltzmann distribution of each conformation.

Gibbs free energies were determined at the B3LYP-D3(BJ)/6-31G* level. Boltzmann weights were calculated using relative Gibbs free energies. DP4+ calculations were performed using the freely available Excel spreadsheet from sarotti-nmr.nmr.weebly.com.

### 4.5. Calculations of ECD Spectra

Conformational searches and DFT calculations were carried out using the Spartan 18 program (Wavefunction Inc., Irvine, CA, USA) and the Gaussian 09 program, respectively. A possible enantiomer of munronpin G (1*S*, 4*S*, 5*R*, 9*S*, 10*R*, 11*S*, 12*R*, 13*R*, 15*S*, 17*R*, 2′*R*, 3′*S*-**1**) was submitted to conformational search at the Molecular Mechanics (MMFF) to give six initial conformers, which were further optimized by DFT calculations at the B3LYP/6-31G(d) level. The stable conformers with Boltzmann distributions over 1% were subjected to TDDFT calculations at the CAMB3LYP/6-31Gþ(d) level in the presence of MeOH with a polarizable continuum model. The resultant rotatory strengths of the lowest 30 excited states for each conformer were converted into Gaussian-type curves with half-bands (0.3 eV) using SpecDis v1.61 [20]. The calculated CD spectrum was composed after correction based on the Boltzmann distribution of the stable conformers.

### 4.6. Derivatization of Sugar Moieties in Compounds ***1*** and ***2***

The absolute configurations of the sugar moieties in compounds **1** and **2** were determined using a chemical derivatization method combined with HPLC analysis, which has been previously established in our research group [14].

The procedure was as follows: Each compound was first subjected to alkaline hydrolysis to remove the side chains. Subsequently, the hydrolysate was reacted with 1 mL of 0.5 mol/L hydrochloric acid in a water bath at 90 °C for 2 h. The reaction mixture was neutralized using IRA 400 anion exchange resin, filtered, and then concentrated under reduced pressure to remove the solvent. To the resulting residue, 0.2 mL of a pyridine solution containing 1 mg of L-cysteine methyl ester hydrochloride was added, and the mixture was incubated at 60 °C for 1 h. After this step, 0.2 mL of a pyridine solution containing 1 mg of o-tolyl isothiocyanate was added to the reaction system, and the incubation was continued at 60 °C for another 1 h to complete the derivatization of the sugar moieties. For comparative analysis, standard samples of L-glucose and D-glucose were derivatized under identical experimental conditions. The derivatized products were analyzed by HPLC under the following parameters: column temperature maintained at 40 °C; mobile phase consisting of 25% acetonitrile in water; detection wavelength set at 254 nm; and a flow rate of 0.8 mL/min. HPLC analysis revealed that the retention time of the sugar derivatives from both compounds **1** and **2** was consistent with that of the D-glucose standard derivative under the same analytical conditions (t_R_ = 9.64 min). Based on this comparison, the sugar moieties in compounds 1 and 2 were identified as D-glucose.

### 4.7. Determination of Inflammatory Mediators in LPS-Induced RAW264.7 Cells

A cell model of inflammatory injury was established by lipopolysaccharide (LPS)-induced murine mononuclear macrophage leukemia RAW 264.7 cells, and the level of nitric oxide (NO) release from cells was detected using the Griess method. The specific procedures were as follows: When the density of RAW 264.7 cells reached 80%, the cells were passaged and seeded into 96-well plates at a density of 5 × 10^3^ cells per well. After 24 h of incubation, drugs were administered, and different groups were set up: blank control group, model group (treated with LPS at 1 μg/mL), positive control group (treated with dexamethasone at 40 and 80 μM combined with LPS at 1 μg/mL), and administration groups (treated with monomer compounds at different concentrations (10, 20, 40, 80 μM) or crude extract fractions at different concentrations combined with LPS at 1 μg/mL). After 24 h of incubation, 50 μL of the culture supernatant was transferred to a new 96-well plate using a 50 μL pipette tip. Then, 50 μL of Griess Reagent I and 50 μL of Griess Reagent II were added to each well respectively. The absorbance (OD value) of each well was measured at a wavelength of 540 nm using a microplate reader. The concentration of nitric oxide in the samples was calculated according to the standard curve.

### 4.8. Cytotoxicity Assay

The cytotoxic activities of the isolated limonoids, munropins G–J (**1**–**4**), were evaluated against four human cancer cell lines (A549, HepG2, MCF-7, and HCT116), using the Cell Counting Kit-8 (CCK-8) (Dojindo Laboratories, Kumamoto, Japan) assay according to the manufacturer’s protocol. The concentration range (5–80 μM) was selected based on preliminary screening results and the literature precedence for limonoid cytotoxicity studies [21], aiming to detect both potent and weak activities; the upper limit of 80 μM represents a practical threshold for significant activity consistent with standard practice in natural product screening, balancing the need to avoid false negatives with considerations of compound solubility and cellular tolerance. Briefly, cells were seeded into 96-well plates at a density of 2 × 10^4^ cells per well in 100 μL of culture medium and allowed to adhere overnight. The cells were then treated with various concentrations of each compound (5–80 µM), while control wells received an equal volume of dimethyl sulfoxide (DMSO) as the vehicle. Following 24 h of incubation, 10 μL of CCK-8 solution was added to each well and incubated for an additional 2 h. The absorbance at 450 nm was measured using a microplate reader, and cell viability (%) was calculated relative to the vehicle-treated control group.

## 5. Conclusions

This phytochemical investigation of the aerial parts of *Munronia pinnata* led to the isolation and characterization of four new limonoids, designated as munropins G–J (**1**–**4**). Their structures, featuring a prieurianin-type skeleton with variations at the C-17 side chain, were conclusively established through comprehensive spectroscopic analysis, quantum chemical calculations (DP4+ and TDDFT-ECD), and chemical derivatization. Notably, the absolute configurations of munropins G (**1**) and H (**2**) were unambiguously determined. Biological evaluation revealed that, at a concentration of 80 µM, compounds **1**–**4** did not exhibit significant cytotoxicity against a panel of human cancer cell lines (A549, HepG2, MCF-7, HCT116) nor potent inhibitory effects on NO production in LPS-induced RAW 264.7 macrophages.

In summary, this study successfully expands the chemical library of the genus Munronia by identifying four novel prieurianin-type limonoids. While the isolated compounds showed limited activity in the specific assays conducted, their structural elucidation provides valuable data for chemotaxonomic studies and offers new molecular scaffolds for future structure–activity relationship exploration and potential synthetic modification to uncover latent bioactivities.

## Figures and Tables

**Table 1:** ^1^H NMR and ^13^C NMR (500/125 MHz, CD_3_OD) data of munropins G–H (**1**–**2**).

Position	1		2	
	δ_C_ (Type)	δ_H_ (*J* in Hz)	δ_C_ (Type)	δ_H_ (*J* in Hz)
1	77.9	4.70 (1H, d, 10.2)	78.4	4.71 (1H, m)
2	37.4	2.36 (1H, dd, 10.1, 16.0)	37.5	2.35 (1H, dd, 16.0, 10.0)
		2.81 (1H, m)		2.84 (1H, d, 14.7)
3	176.3	-	173.5	-
4	84.1	-	84.1	-
5	46.1	2.76 (1H, m)	45.7	2.99 (1H, m)
6	32.5	2.51 (1H, m)	32.9	2.45 (1H, dd, 16.9, 11.9)
		2.99 (1H, m)		2.99 (1H, m)
7	176.9	-	177.5	-
8	132.4	-	133.1	-
9	44.9	3.15 (1H, d, 5.7)	47.2	3.12 (1H, d, 11.5)
10	53	-	52.8	-
11	73.4	5.54 (1H, m)	72.8	4.19 (1H, dd, 9.8, 5.2)
12	76.5	5.54 (1H, m)	78.6	5.47 (1H, d, 9.8)
13	52.5	-	51.5	-
14	153.4	-	152.5	-
15	70.4	4.99 (1H, d, 8.2)	70.5	4.96 (1H, d, 8.0)
16	41.2	1.89 (1H, ddd, 14.2, 7.9, 1.5)	41.1	1.87 (1H, dd, 13.9, 7.8)
		2.58 (1H, ddd, 17.1, 9.7, 6.0)		2.53 (1H, m)
17	43.2	3.43 (1H, m)	44.2	3.35 (1H, m)
18	18.4	1.09 (3H, s)	17.8	0.99 (3H, s)
19	18.1	1.05 (3H, s)	18.5	1.08 (3H, s)
20	125.2	-	125	-
21	141.9	7.45 (1H, s)	142.2	7.36 (1H, s)
22	112	6.44 (1H, d, 1.83)	112	6.37 (1H, s)
23	143.9	7.46 (1H, d, 1.83)	143.7	7.39 (1H, s)
28	69.3	3.38 (1H, m)	69.6	3.41 (1H, d, 12.0)
		3.72 (1H, d)		3.68 (1H, d, 11.7)
29	20.8	1.19 (3H, s)	20.9	1.17 (3H, s)
30	65.2	4.21 (1H, m)	63.7	4.24 (1H, m)
		4.59 (1H, d, 13.6)		4.62 (1H, d, 13.1)
7-OAc	171.9	-	-	-
	22.0	2.08 (3H, s)	-	-
1′	173.4	-	172.1	-
2′	83.1	3.44 (1H, m)	84.7	3.75 (1H, d, 5.1)
3′	41.2	1.67 (1H, ddq, 10.2, 7.2, 3.3)	39	1.64 (1H, m)
4′	25.3	1.15 (2H, m)	25.2	1.17 (1H, m)
				1.47 (1H, m)
5′	12.3	0.78 (3H, t, 7.5)	11.9	0.82 (3H, t, 7.4)
6′	15.6	0.84 (3H, d, 6.9)	15	0.78 (3H, d, 6.5)
1″	104.9	4.21 (1H, d, 7.9)	105.5	4.21 (1H, d, 7.9)
2″	74.7	3.25 (1H, m)	75.1	3.22 (1H, m)
3″	77.8	3.38 (1H, m)	77.8	3.35 (1H, m)
4″	78.1	3.38 (1H, m)	78.6	3.35 (1H, m)
5″	71.2	3.38 (1H, m)	71.3	3.35 (1H, m)
6″	62.4	3.80 (1H, m)	62.4	3.75 (1H, m)
		4.00 (1H, m)		3.90 (1H, d, 11.1)

**Table 2:** ^1^H NMR and ^13^C NMR (500/125 MHz, CD_3_OD) data of munropins I–J (**3**–**4**).

Position	3		4	
	δ_C_	δ_H_ (*J* in Hz)	δ_C_	δ_H_ (*J* in Hz)
1	150.1	6.99 (d, 10.4)	150	6.98 (d, 10.7)
2	123.4	6.23 (d, 10.4)	123.4	6.22 (d, 10.7)
3	168.4	-	168.7/168.4	-
4	86.2	-	86.3/86.2	-
5	51.7	3.47 (dd, 6.8, 2.4)	51.6	3.76 (dd, 8.3, 13.4)
6	35.3	2.52 (2H, m)	35.2/35.2	2.51/2.47 (each, m)
7	175.4	-	174.5	-
8	136.8	-	136.5	-
9	54.3	3.20 (d, 7.2)	54	3.21 (dd, 2.1, 7.0)
10	47.3	-	47.2	-
11	71.6	5.74 (dd, 7.2, 10.6)	71.4	5.78 (dd, 7.0, 10.6)
12	76.2	5.89 (d, 10.6)	75.9/76.1	5.92 (d, 11.24)/5.87 (d, 12.1)
13	47.4	-	47.3	-
14	72.1	-	72.1	-
15	61.1	4.05 (brs)	61	4.07 (brs)
16	32.4	2.15, 2.23 (each, m)	32.6	2.04, 2.35 (each, m)
17	39.3	2.87 (br)	41.3	2.79 (dd, 6.8, 10.3)
18	13.5	1.08 (3H, s)	13.6	1.15 (3H, s)
19	24.3	1.16 (3H, s)	24.4	1.04 (3H, s)
20	134.6	-	169.3	-
21	173.4	-	101.8/101.0	5.99/5.86 (1H, s)
22	149.5	7.04 (brs)	121.0/119.7	6.07/5.93 (brs)
23	98.5/98.3	6.02 (brs)	172.2	-
28	24.7	1.33 (3H, s)	24.7	1.35 (3H, s)
29	64.4	4.21, 5.01 (each, d, 11.6)	64.4	4.21, 5.01 (each, d, 12.6)
30	123.7	5.50, 5.52 (each, brs)	123.4	5.52, 5.53 (each, brs)
OMe-7	52.8	3.71 (3H, s)	52.82	3.70 (3H, s)
OAc-28	20.5	2.06 (3H, s)	20.5	2.08 (3H, s)
	161.6	-	161.5	-
OCOH-11	174.5	8.06 (d, 2.27)	173.1	8.07 (d, 5.9)
2′	76.4	3.80 (dd, 3.8, 6.8)	76.6	3.84/3.77 (d, 4.3)
3′	39.2	1.58 (m)	39.3	1.61 (m)
4′	24.1	1.28/1.162 (m)	24	1.17 (m)
5′	15.8	0.84 (3H, dd, 7.2, 8.1)	11.8/11.5	0.88 (dd, 6.9, 17.0)
6′	11.7	0.86 (3H, d, 6.8)	15.6	0.85 (3H, d, 6.8)

## Data Availability

The original contributions presented in this study are included in the article/Supplementary Materials. Further inquiries can be directed to the corresponding authors.

## Supplementary Materials

The following supporting information can be downloaded at: https://www.mdpi.com/article/10.3390/ijms27073331/s1.

## Abbreviations

The following abbreviations are used in this manuscript:

A549	human lung adenocarcinoma cell line
Bax	Bcl-2-associated X protein
Bcl-2	B-cell lymphoma 2
CCK-8	Cell Counting Kit-8
CD_3_OD	deuterated methanol
COSY	correlation spectroscopy
DEPT	enhancement by polarization transfer
DFT	density functional theory
DMSO	dimethyl sulfoxide
ECD	electronic circular dichroism
EC_50_	half maximal effective concentration
GIAO	gauge-independent atomic orbital
GPC	gel permeation chromatography
HCT116	colorectal carcinoma cell line
HepG2	hepatocellular carcinoma cell line
HMBC	heteronuclear multiple bond correlation
HPLC	high-performance liquid chromatography
HRESIMS	electrospray ionization mass spectrometry
HSQC	heteronuclear single quantum coherence
HSCCC	speed counter-current chromatography
IR	infrared
LD_50_	median lethal dose
LPS	lipopolysaccharide
MCF-7	breast adenocarcinoma cell line
MeOH	methanol
MIC	minimum inhibitory concentration
MMFF	Merck Molecular Force Field
NMR	nuclear magnetic resonance
NO	nitric oxide
ODS	octadecylsilyl
PAL	phenylalanine ammonia-lyase
PCM	polarizable continuum model
POD	peroxidase
PPO	polyphenol oxidase
PR-1a	pathogenesis-related protein 1a
PR-5	pathogenesis-related protein 5
RAW	murine macrophage cell line
ROESY	frame Overhauser effect spectroscopy
RP-C18	reversed-phase C18
SAR	Structure–activity relationship
SOD	superoxide dismutase
TDDFT	dependent density functional theory
TMS	tetramethylsilane
TMV	tobacco mosaic virus
