# Supplementary material for: Munropins G–J: Four New Prieurianin-Type Limonoids from Munronia pinnata and Their Structural and Molecular Characterization
Source: Int J Mol Sci. 2026 Apr 7;27(7):3331. doi: 10.3390/ijms27073331 (PMC13073901; doi:10.3390/ijms27073331)
Supplement: Supplementary file 1 [file ijms-27-03331-s001.zip › ijms-4153092-supplementary.pdf]

Munropins G–J: Four New Prieurianin-Type Limonoids from *Munronia pinnata* and Their Structural and Molecular Characterization

Xuerong Yang [1,†], Jianxing Li [1,†], Peiyuan Liu [2], Xiaojie Yan [2], Fenglai Lu [2], Yoshiki Kashiwada [3], Xiangqin Li [4], Naonobu Tanaka [3,*] and Dianpeng Li [5,*]

[1] School of Pharmacy, Guangxi Health Science College, Nanning 530023, China
[2] Guangxi Key Laboratory of Plant Functional Phytochemicals and Sustainable Utilization, Guangxi Institute of Botany, Guangxi Zhuang Autonomous Region and Chinese Academy of Sciences, Guilin 541006, China
[3] Graduate School of Pharmaceutical Sciences, Tokushima University, Tokushima 770-8505, Japan
[4] Guangxi Key Laboratory of Plant Conservation and Restoration Ecology in Karst Terrain, Guangxi Institute of Botany, Guangxi Zhuang Autonomous Region and Chinese Academy of Sciences, Guilin 541006, China
[5] Guangxi Academy of Sciences, Nanning 530007, China
* Correspondence: ntanak@tokushima-u.ac.jp (N.T.); phytoldp@hotmail.com (D.L.)
† These authors contributed equally to this work.

Contents of Supporting Information

Figure S1. Key 2D-NMR correlations of munropins H-J (**2-4**)........2
Table S1. Inhibitory effect of munropins G-J (**1-4**) on tumor cell proliferation 2
Figure S2. HRESIMS of compound **1**........3
Figure S3. ^{1}H-NMR spectrum of compound **1** in CD_3OD.
Figure S4. DEPTQ spectrum of compound **1** in CD_3OD........5
Figure S5. ^{1}H-^{1}H COSY spectrum of compound **1** in CD_3OD........6
Figure S6. HSQC spectrum of compound **1** in CD_3OD........7
Figure S7. HMBC spectrum of compound **1** in CD_3OD........8
Figure S8. ROESY spectrum of compound **1** in CD_3OD........9
Figure S9. IR spectrum of compound **1**........10
Figure S10. HRESIMS of compound **2**........11
Figure S11. ^{1}H-NMR spectrum of compound **2** in CD_3OD.
Figure S12. DEPTQ spectrum of compound **2** in CD_3OD........13
Figure S13. ^{1}H-^{1}H COSY spectrum of compound **2** in CD_3OD........14
Figure S14. HSQC spectrum of compound **2** in CD_3OD........15
Figure S15. HMBC spectrum of compound **2** in CD_3OD........16
Figure S16. ROESY spectrum of compound **2** in CD_3OD........17
Figure S17. ECD of compound **2**........18
Figure S18. HRESIMS of compound **3**........19
Figure S19. ^{1}H-NMR spectrum of compound **3** in CD_3OD........20
Figure S20. ^{13}C spectrum of compound **3** in CD_3OD........21
Figure S21. ^{1}H-^{1}H COSY spectrum of compound **3** in CD_3OD........22
Figure S22. HSQC spectrum of compound **3** in CD_3OD........23
Figure S23. HMBC spectrum of compound **3** in CD_3OD........24
Figure S24. ROESY spectrum of compound **3** in CD_3OD........25
Figure S25. HRESIMS of compound **4**........26
Figure S26. ^{1}H-NMR spectrum of compound **4** in CD_3OD.
Figure S27. ^{13}C spectrum of compound **4** in CD_3OD........28
Figure S28. ^{1}H-^{1}H COSY spectrum of compound **4** in CD_3OD........29
Figure S29. HSQC spectrum of compound **4** in CD_3OD........30
Figure S30. HMBC spectrum of compound **4** in CD_3OD........31
Figure S31. ROESY spectrum of compound **4** in CD_3OD........32

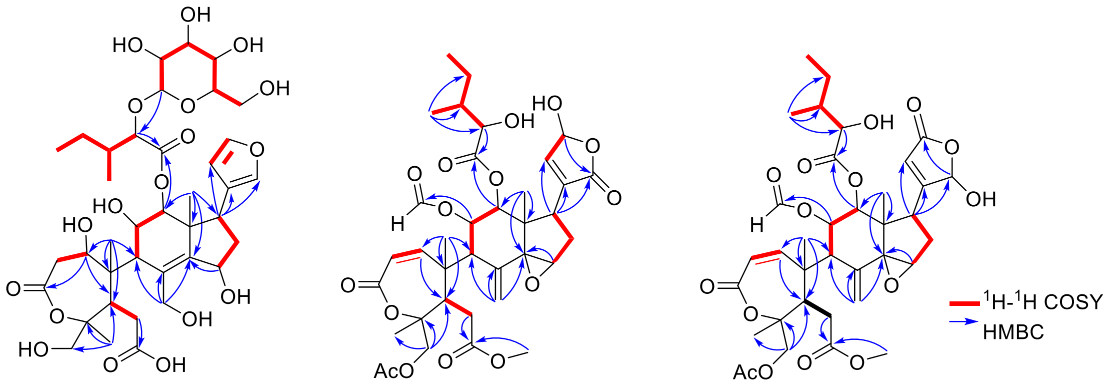

2 **3** **4**

Figure S1. Key 2D-NMR correlations of munropins H–J (**2–4**).

Table S1. Inhibitory effect of munropins G–J (**1–4**) on tumor cell proliferation

compounds	IC$_{50}$ (μM) 72h				
	A549	HepG2	MCF-7	MH7A	HCT116
**1**	>80	>80	>80	>80	>80
**2**	>80	>80	>80	>80	>80
**3**	>80	>80	>80	>80	>80
**4**	>80	>80	>80	>80	>80

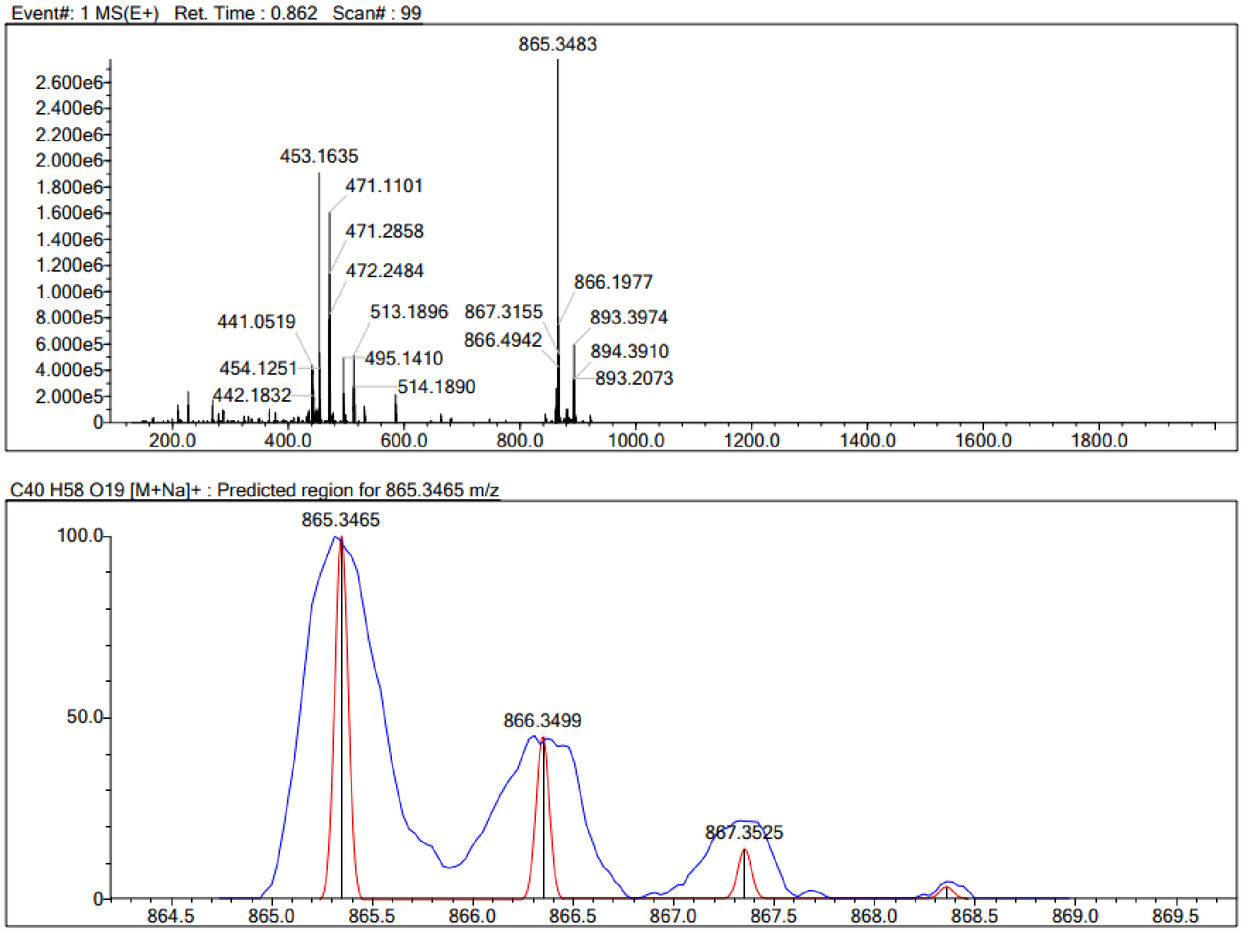

Rank	Score	Formula (M)	Ion	Meas. m/z	Pred. m/z	Df. (mDa)	Df. (ppm)	Iso	DBE	Meas. (M)	Pred. (M)
1	33.79	C40 H58 O19	[M+Na]+	865.3483	865.3465	1.8	2.08	34.73	12.0	842.3591	842.3572

Figure S2. HRESIMS of compound **1**.

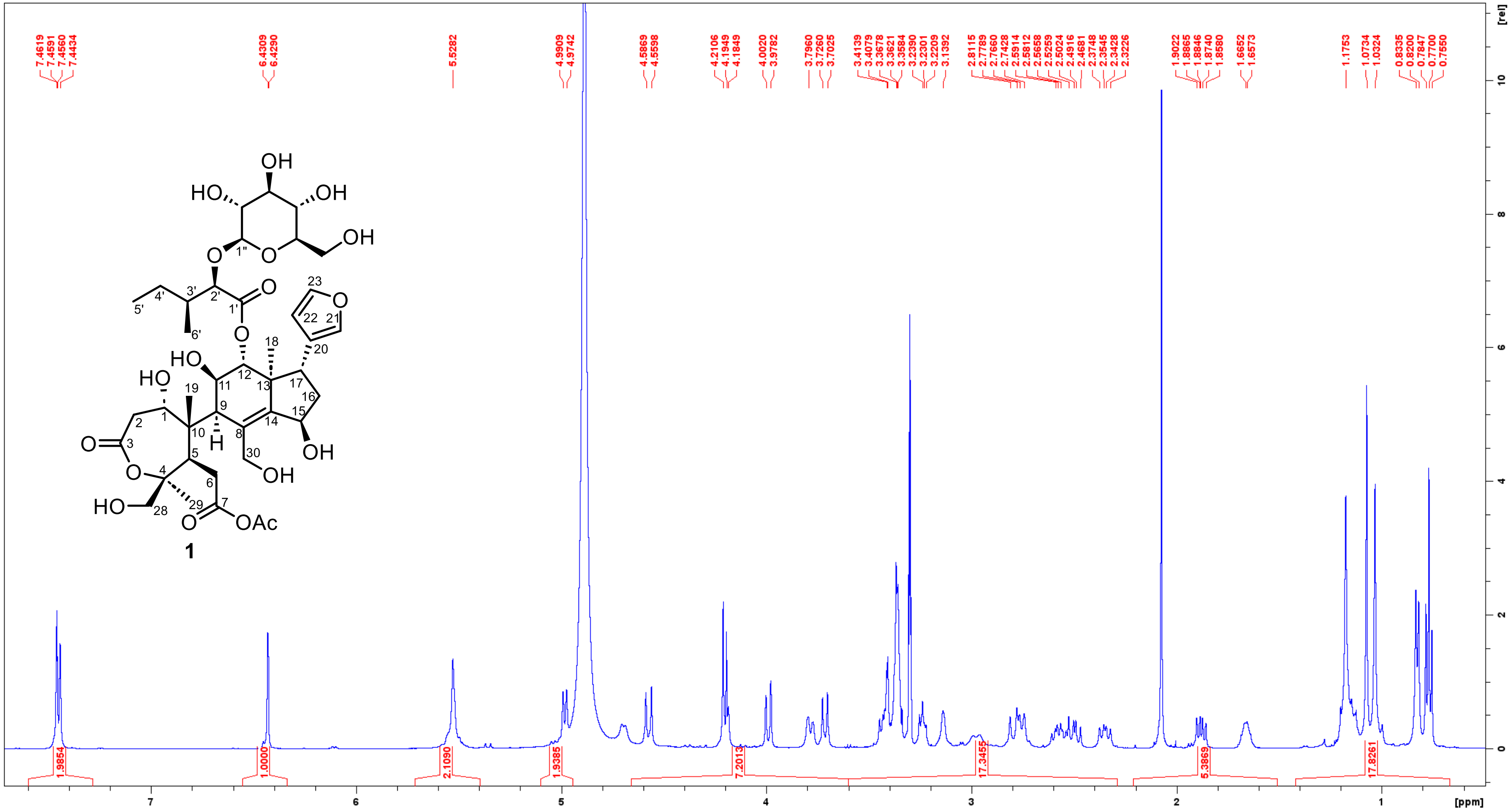

Figure S3. ^{1}H-NMR spectrum of compound **1** in CD_3OD.

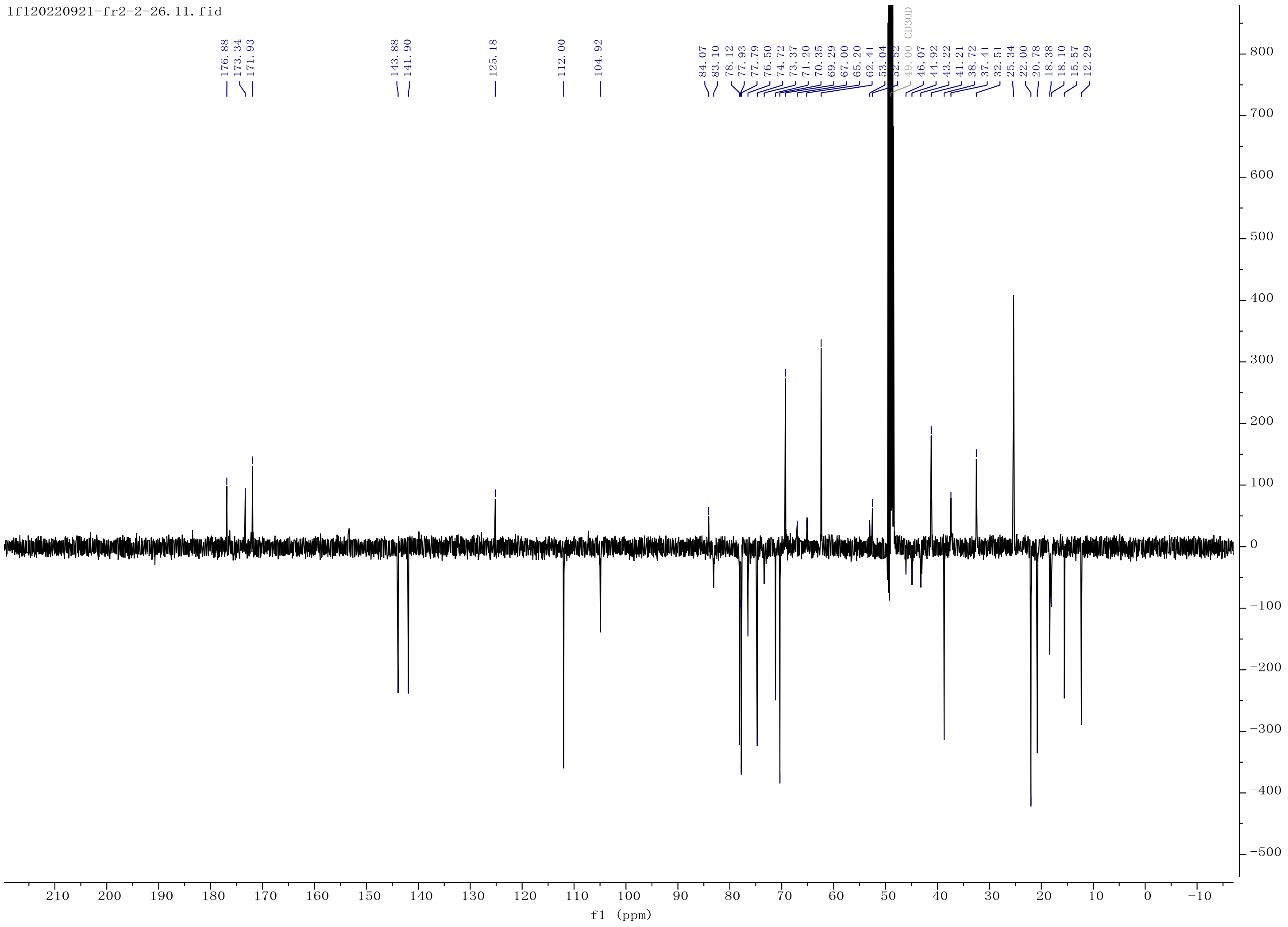

Figure S4. DEPTQ spectrum of compound **1** in CD_3OD.

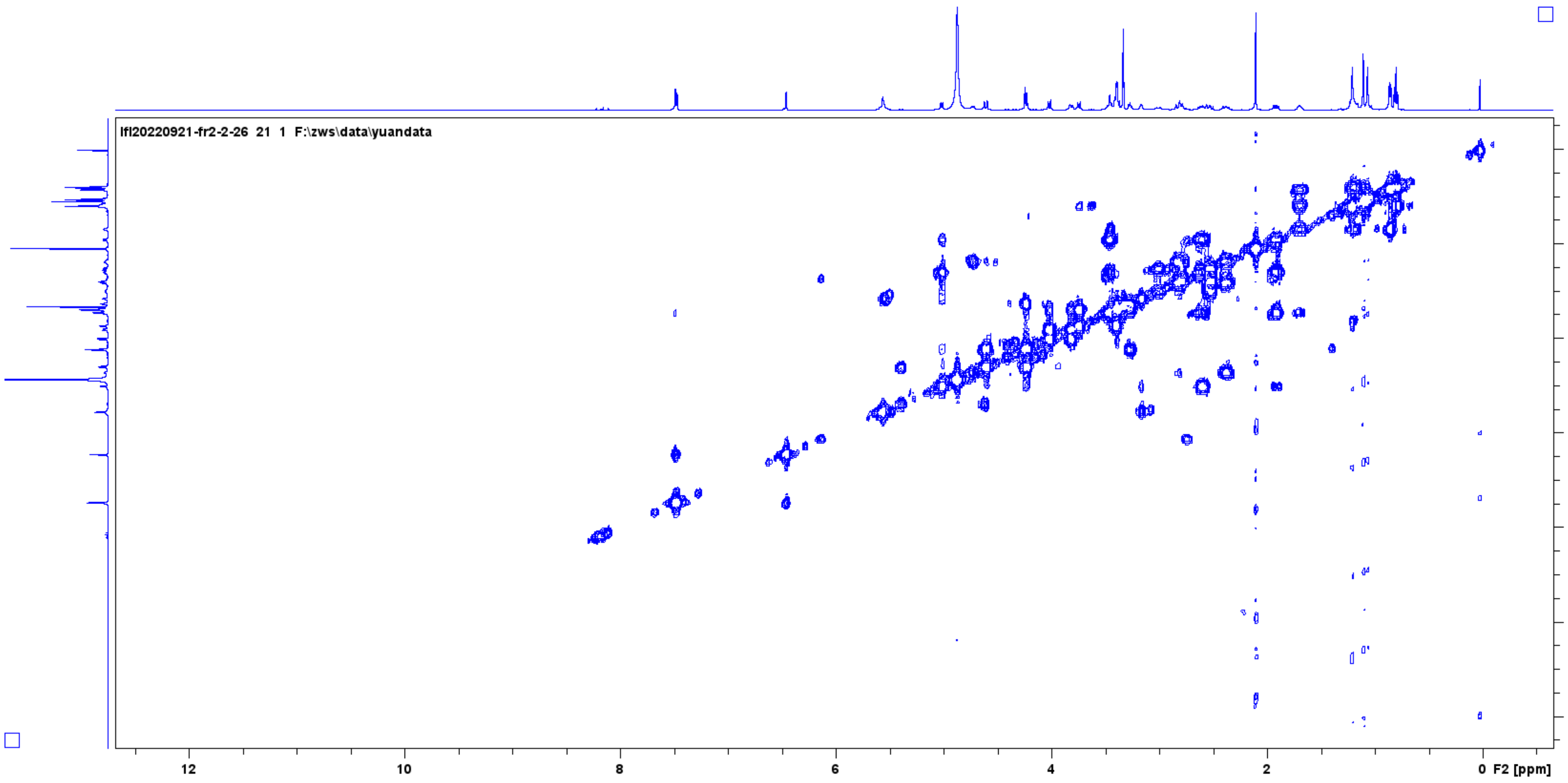

Figure S5. ^{1}H-^{1}H COSY spectrum of compound **1** in CD_3OD.

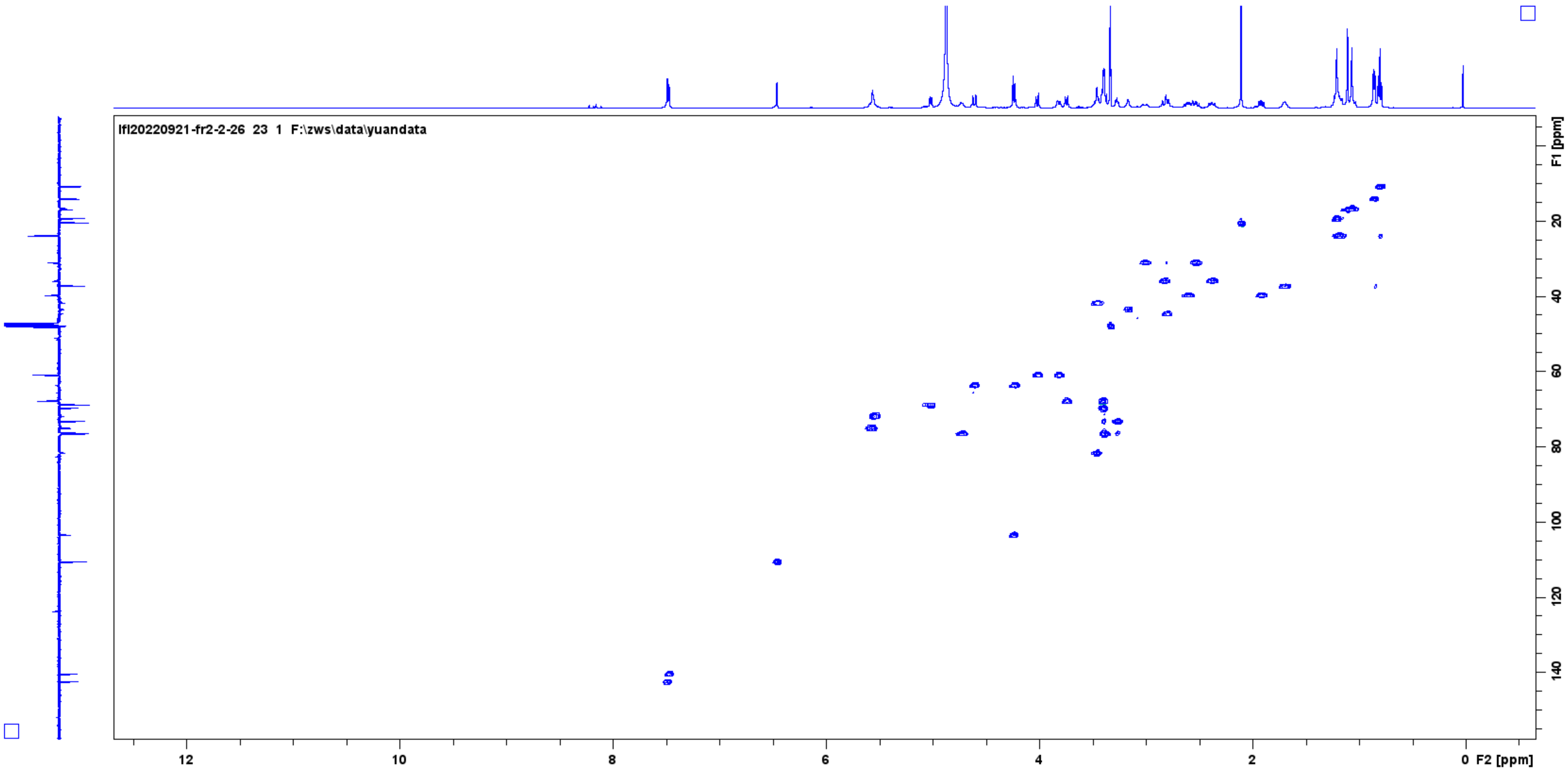

Figure S6. HSQC spectrum of compound **1** in CD_3OD.

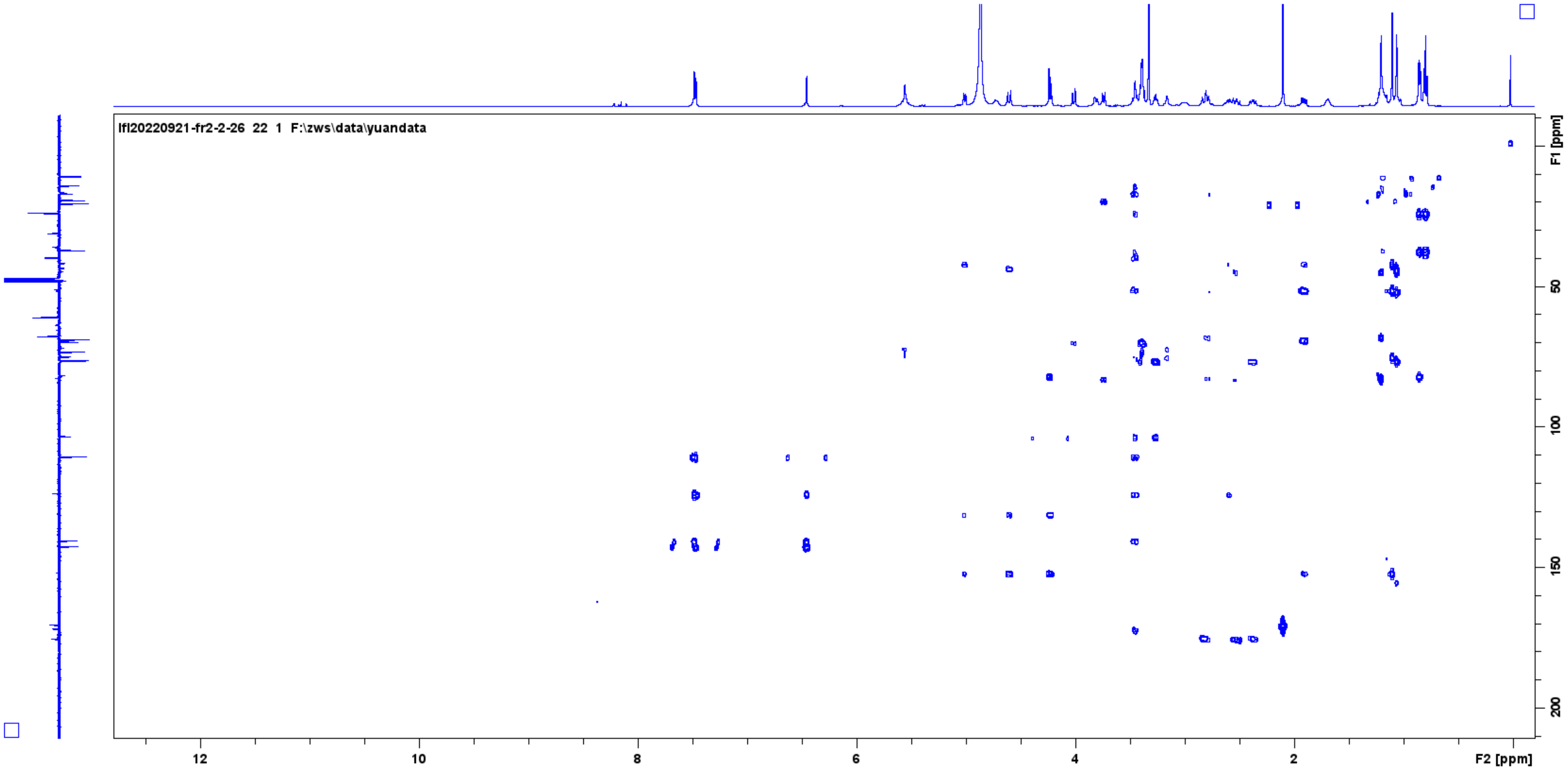

Figure S7. HMBC spectrum of compound **1** in CD_3OD.

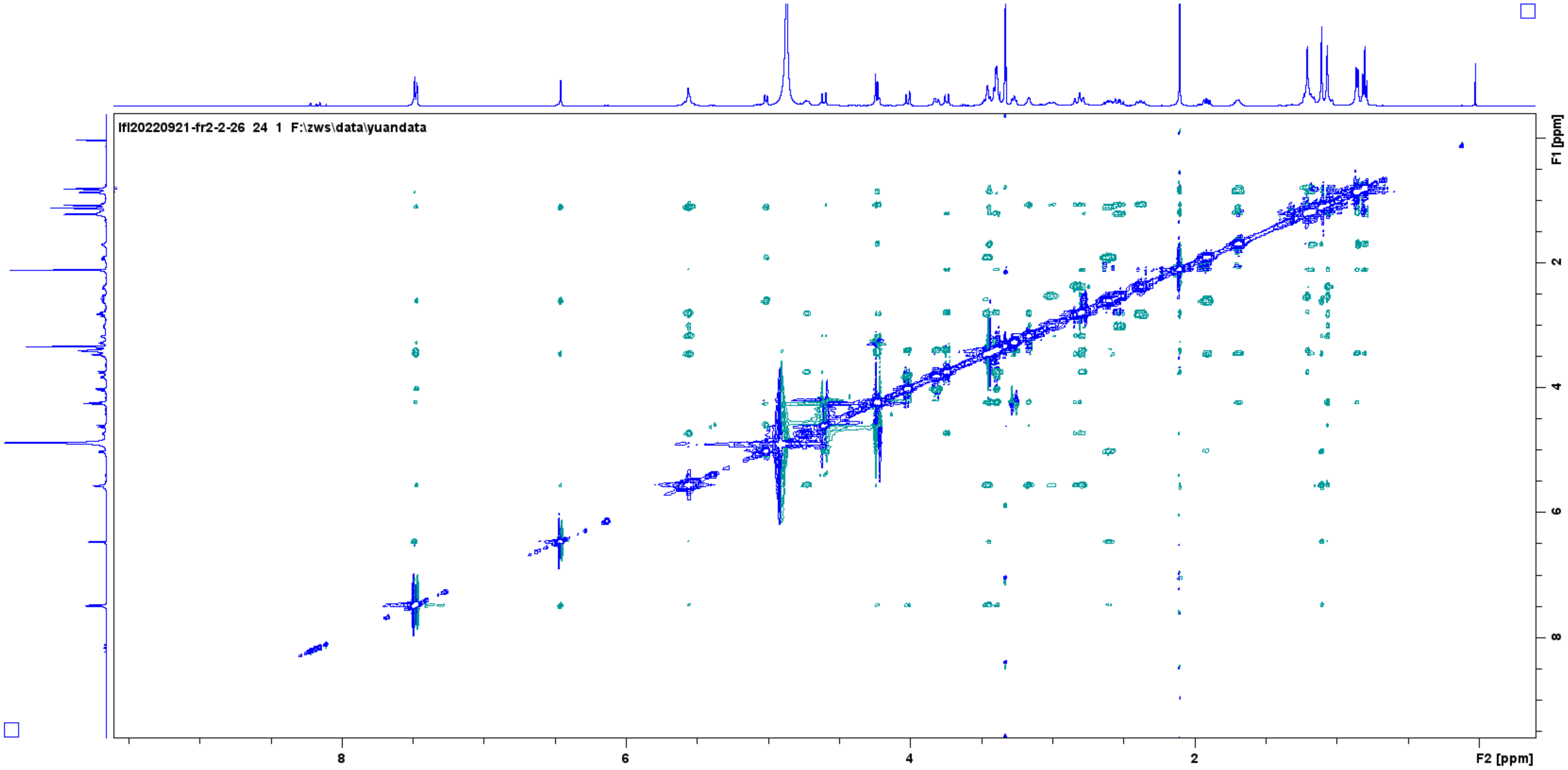

Figure S8. ROESY spectrum of compound **1** in CD_3OD.

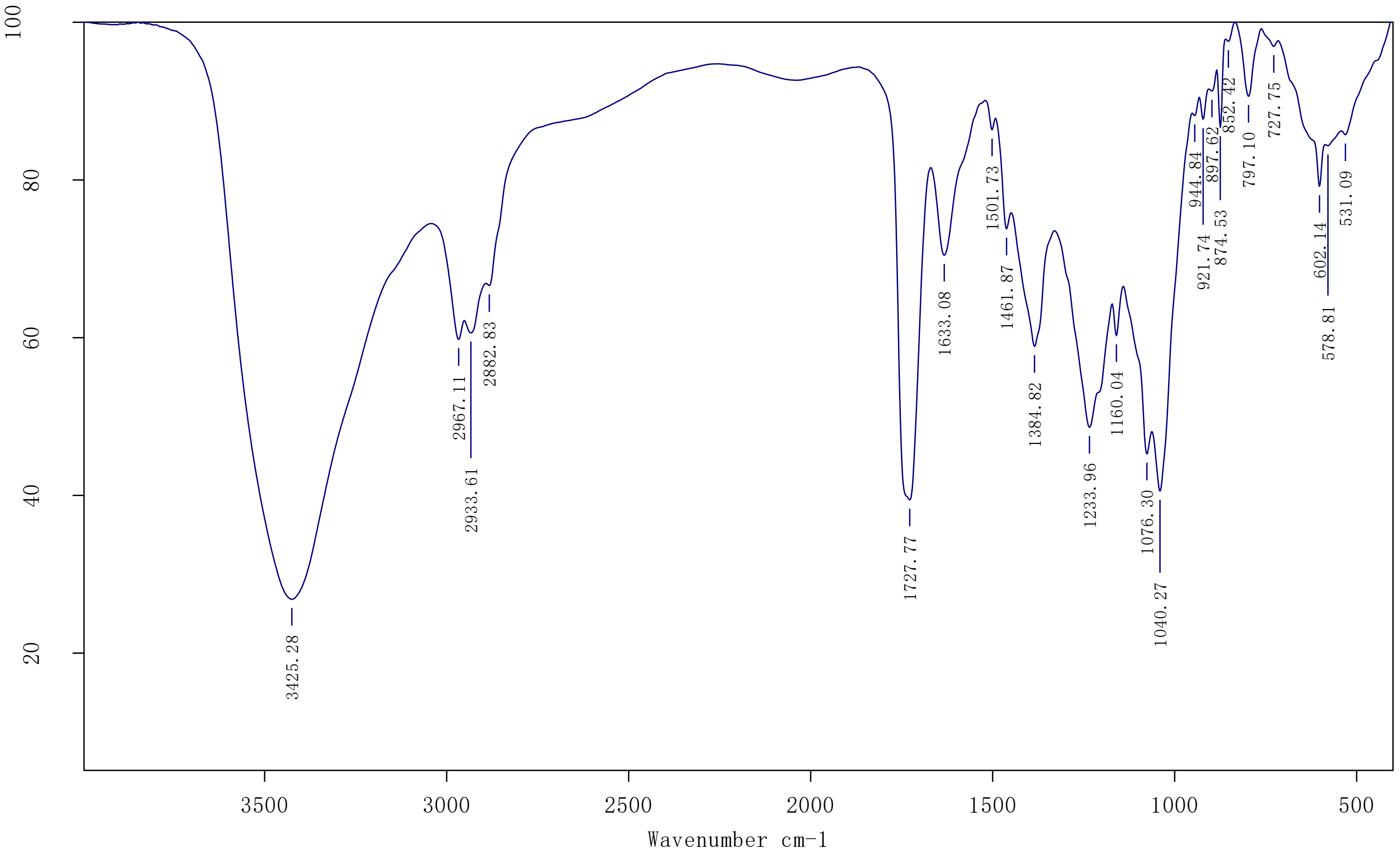

Sample Name: 26
Sample Form: KBr
Path of File: E:\data
Date of Measurement: 2023/1/17

Resolution: 4
Aperture Setting: 6 mm
Number of Background Scans: 16
Number of Sample Scans: 16

Beamsplitter Setting: KBr
Source Setting: MIR
Instrument Type: BRUKER VERTEX 70
Soft Version: OPUS8.1

Figure S9. IR spectrum of compound **1**.

Event#: 1 MS(E+) Ret. Time : 0.353 -> 0.735 Scan# : 43 -> 85

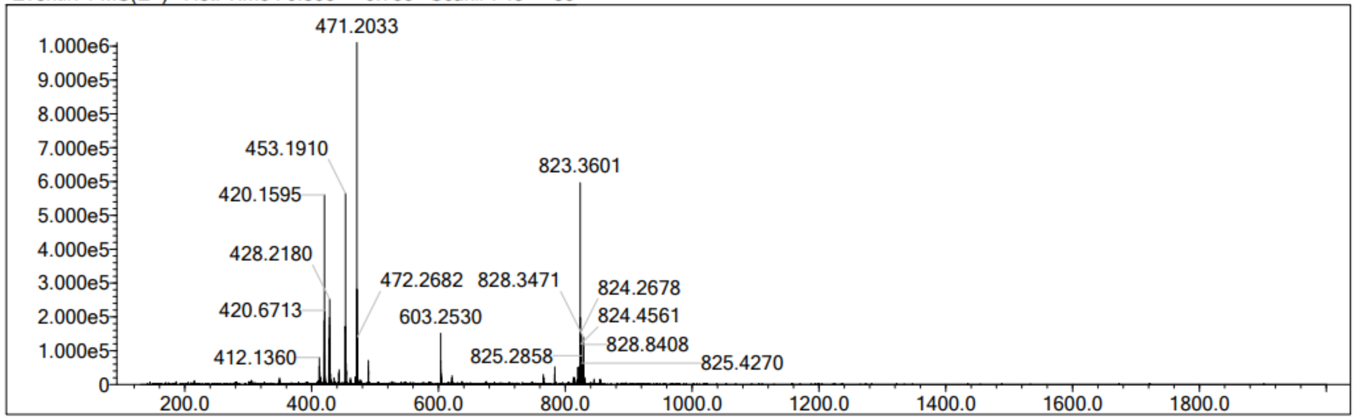

C42 H56 O15 [M+Na]+ : Predicted region for 823.3511 m/z

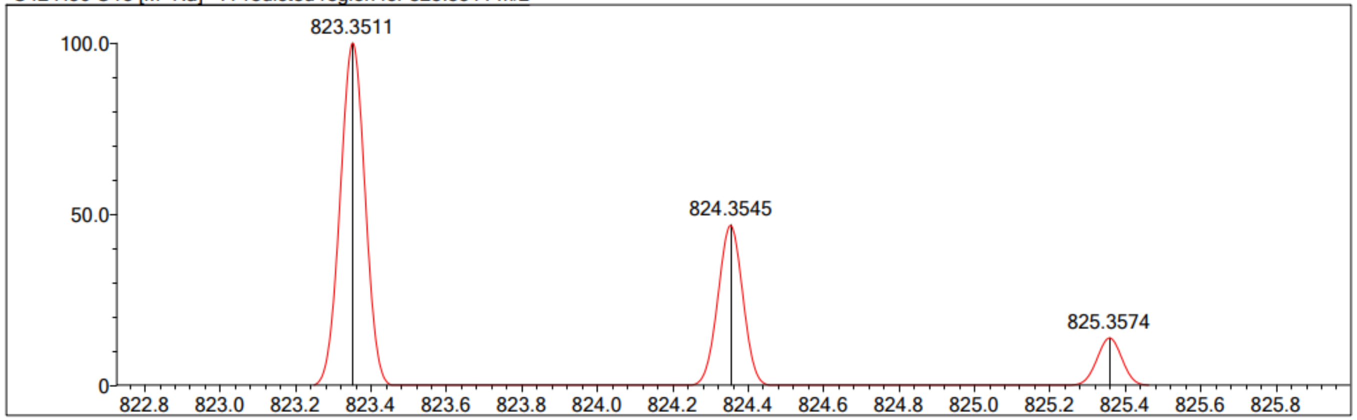

Rank	Score	Formula (M)	Ion	Meas. m/z	Pred. m/z	Df. (mDa)	Df. (ppm)	Iso	DBE	Meas. (M)	Pred. (M)
4	0.84	C42 H56 O15	[M+Na]+	823.3601	823.3511	9.0	10.93	2.25	15.0	800.3709	800.3619

Figure S10. HRESIMS of compound **2**.

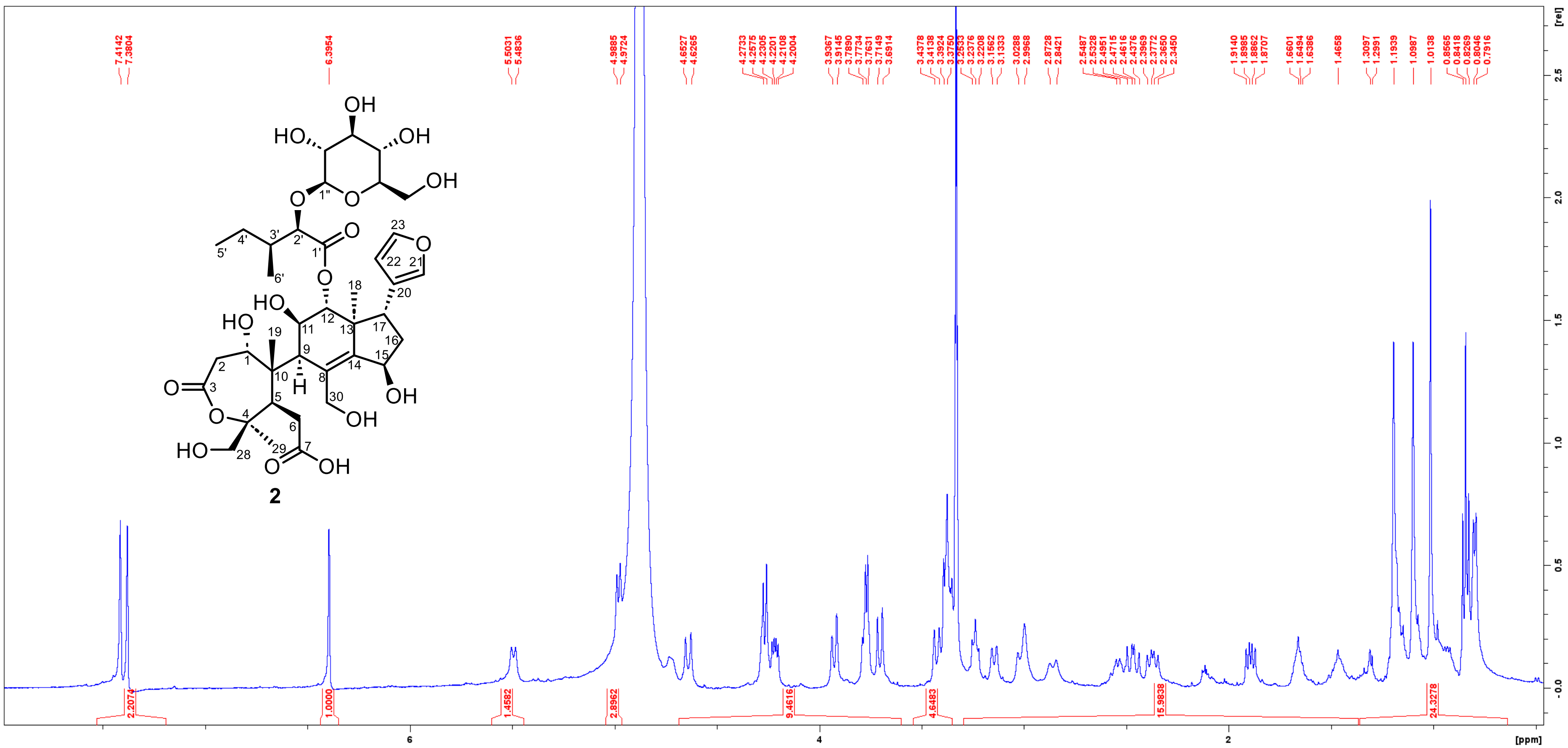

Figure S11. ^{1}H-NMR spectrum of compound **2** in CD_3OD.

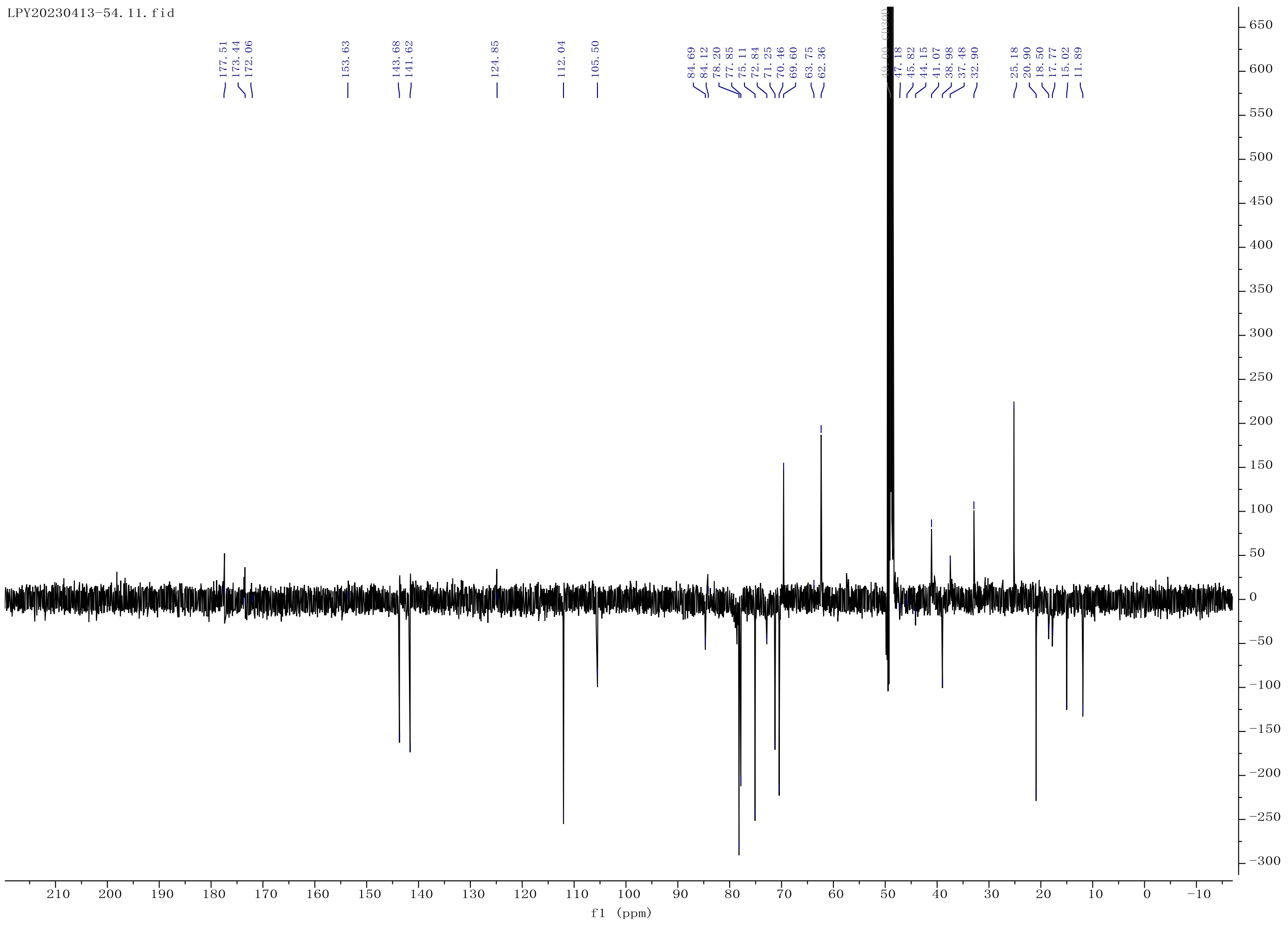

Figure S12. DEPTQ spectrum of compound **2** in CD_3OD.

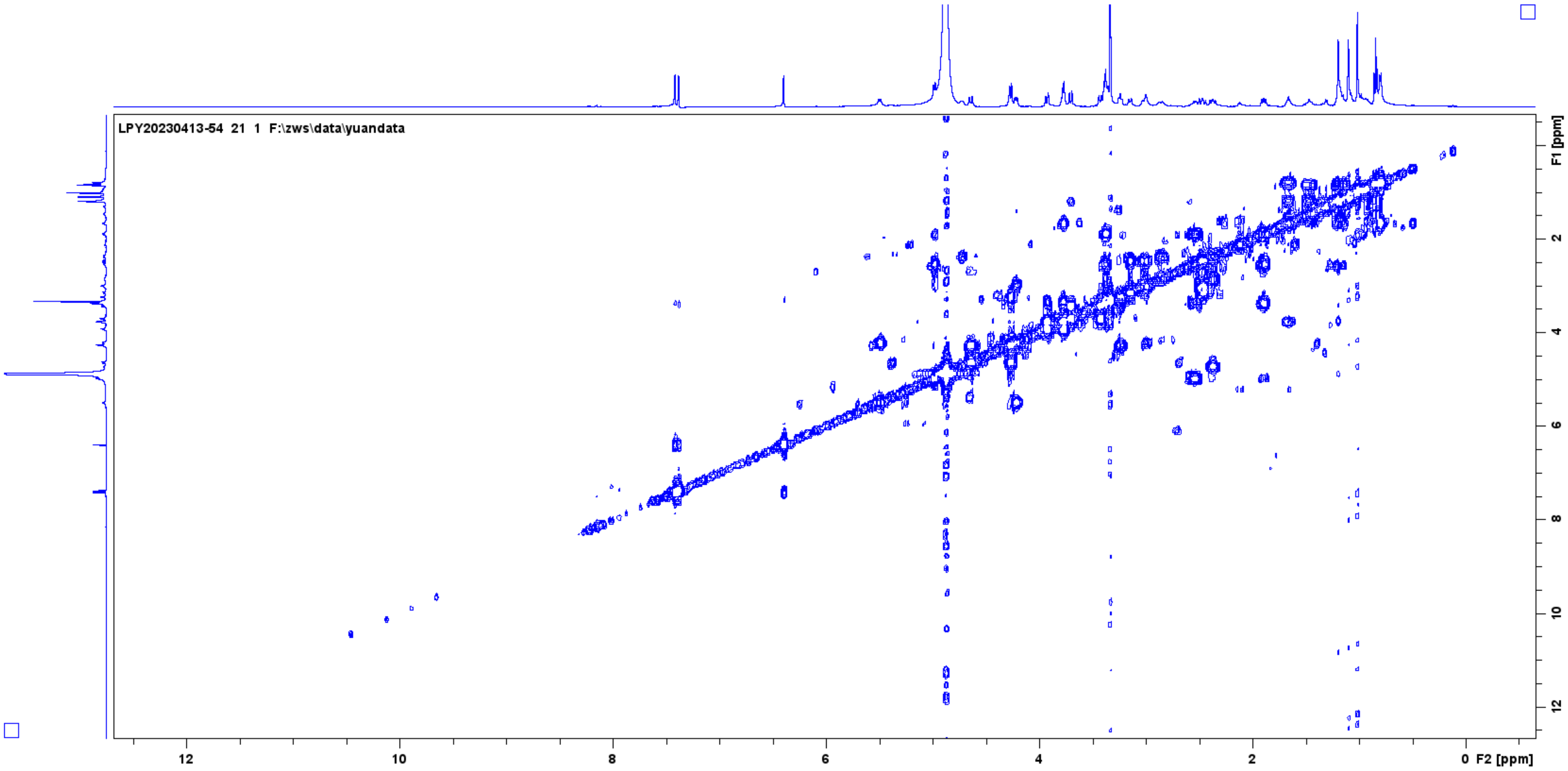

Figure S13. ^1H-^1H COSY spectrum of compound **2** in CD_3OD.

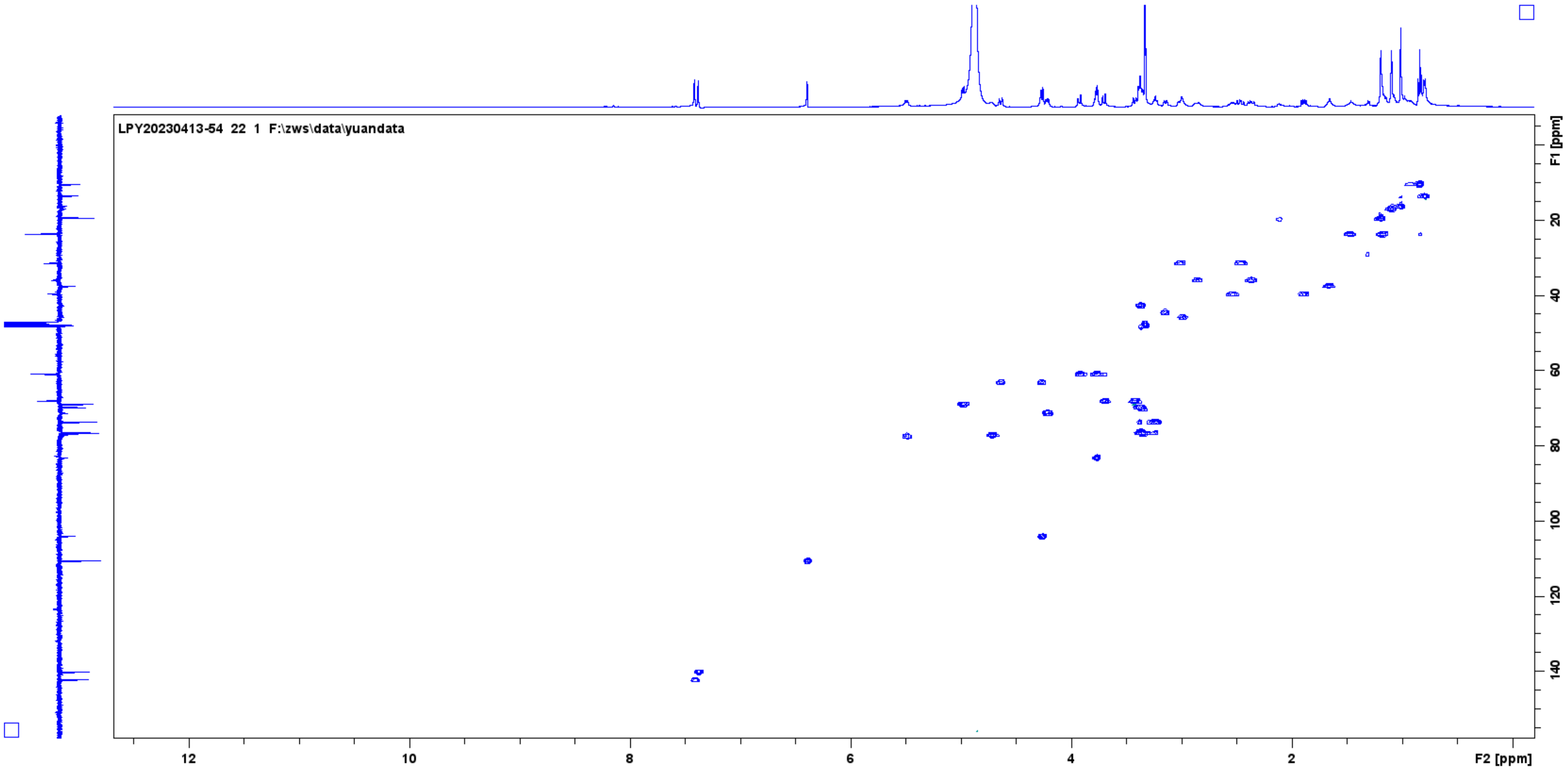

Figure S14. HSQC spectrum of compound **2** in CD_3OD.

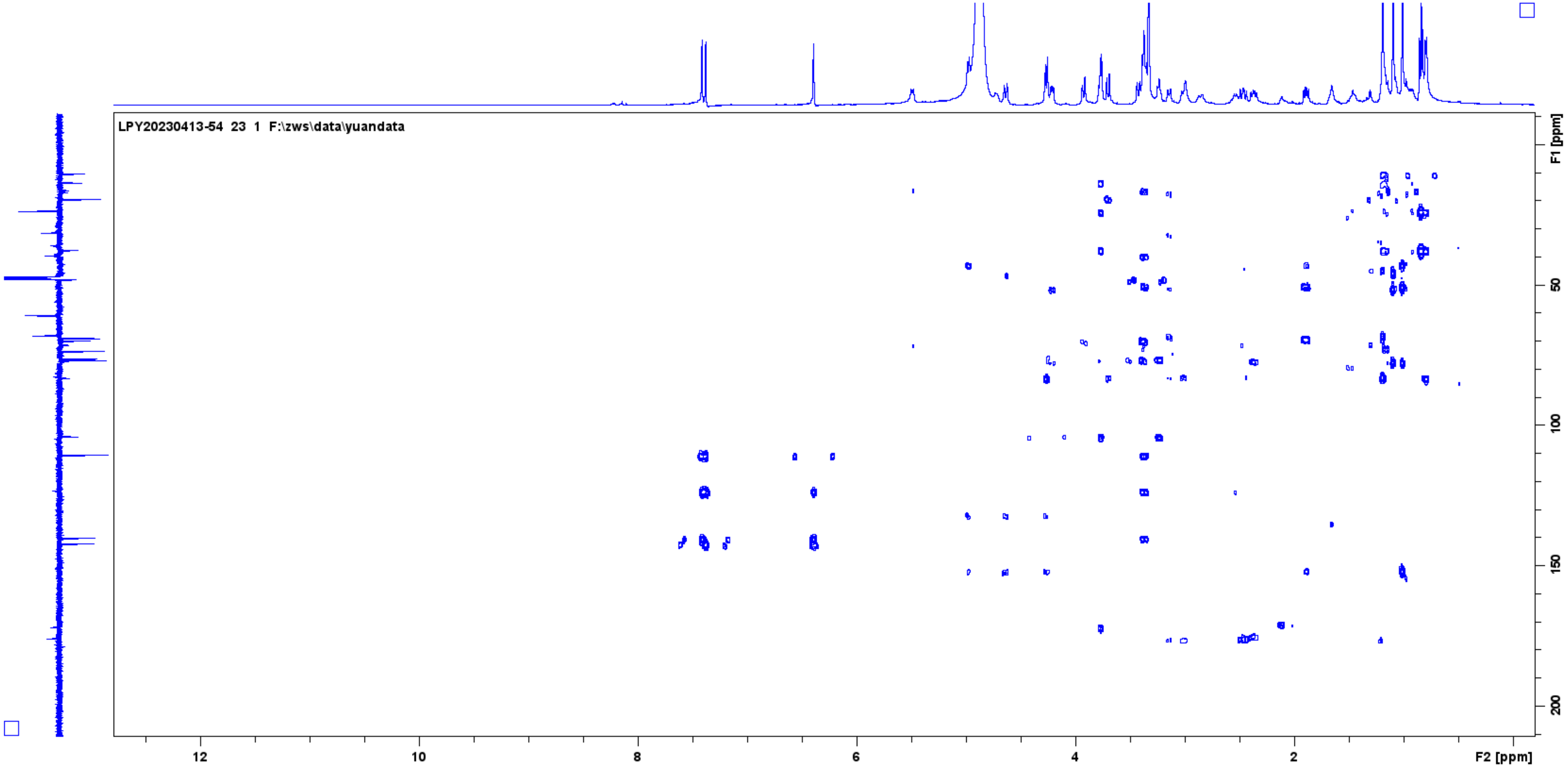

Figure S15. HMBC spectrum of compound **2** in CD_3OD.

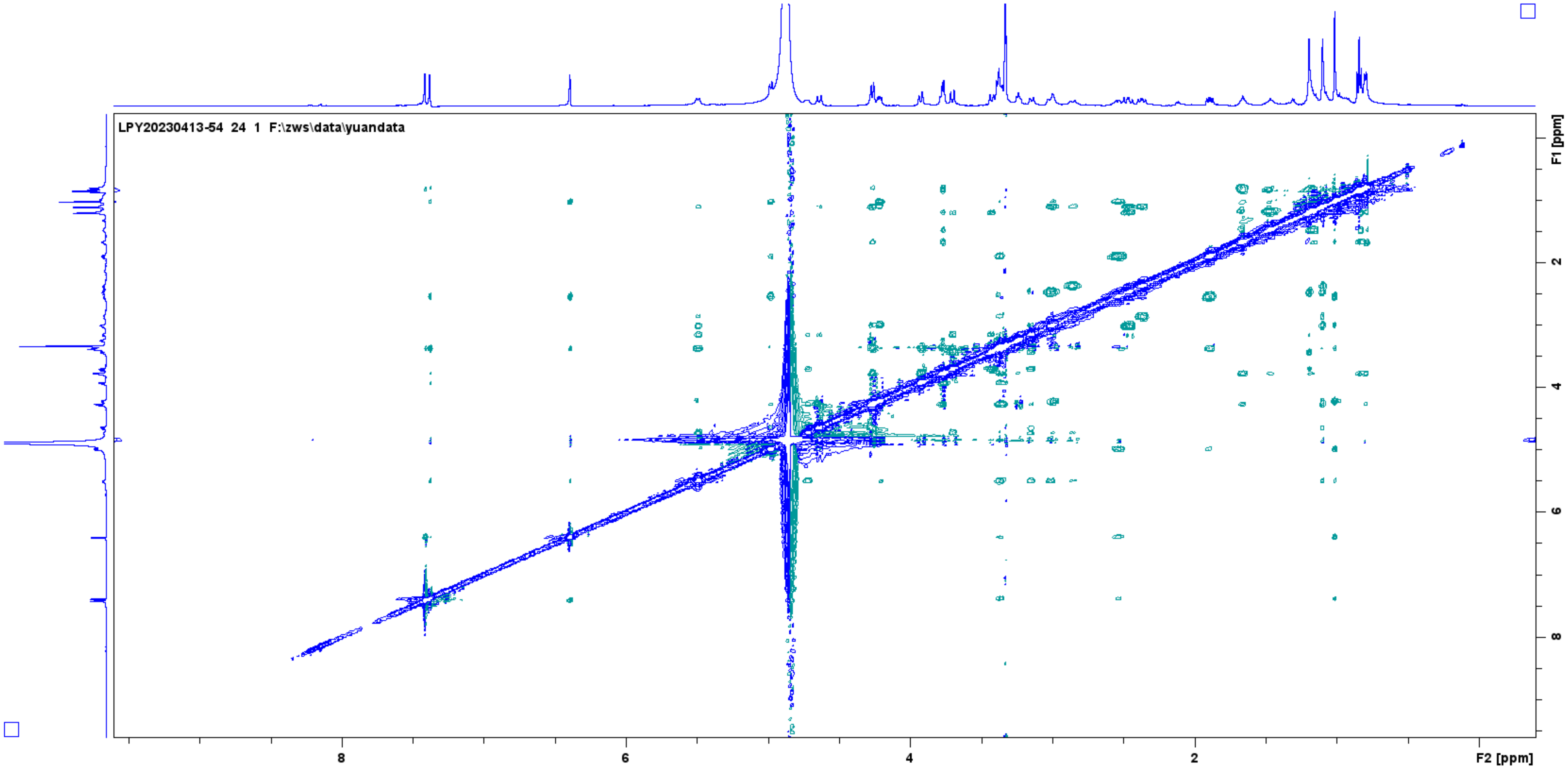

Figure S16. ROESY spectrum of compound **2** in CD_3OD.

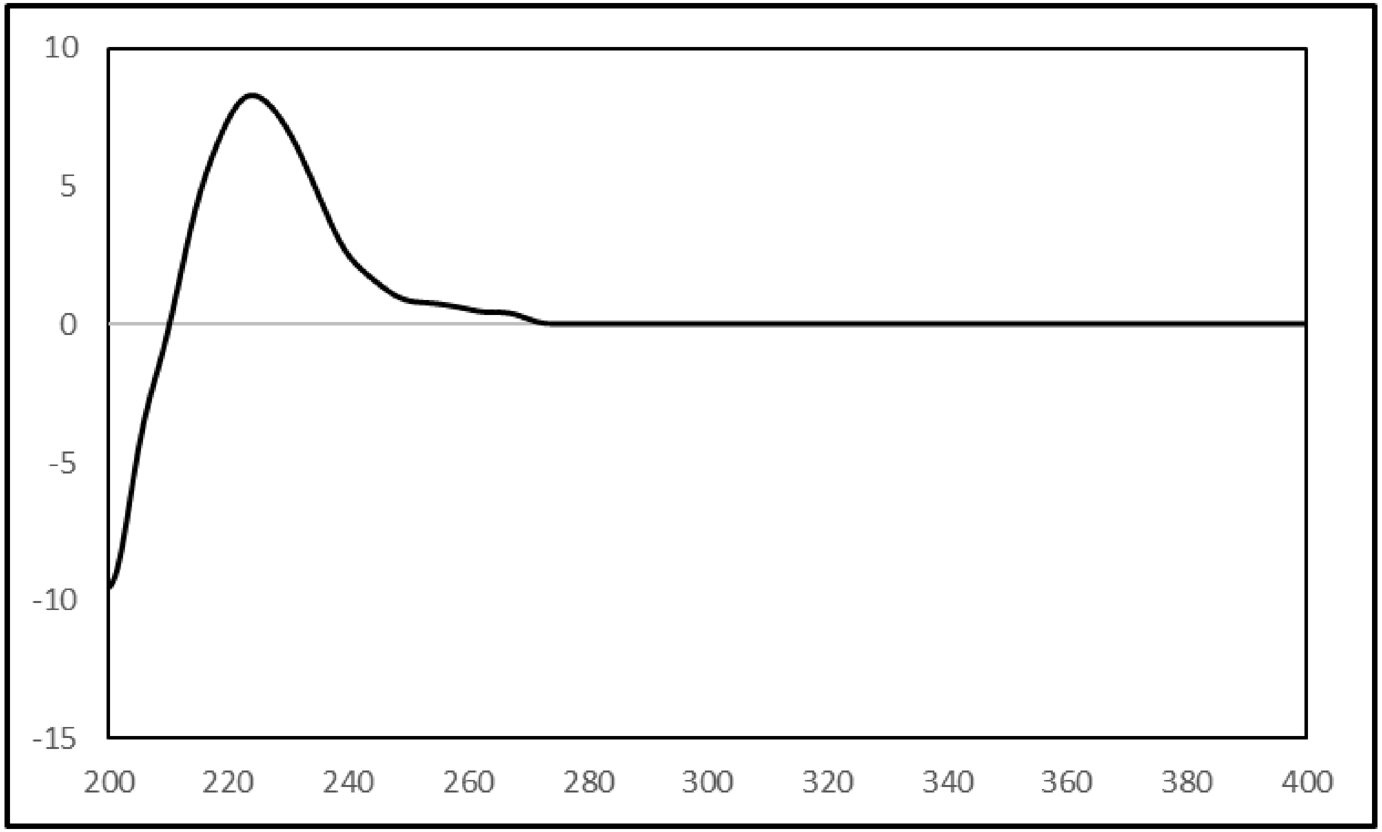

Figure S17. ECD of compound **2**.

Single Mass Analysis
Tolerance = 100.0 PPM / DBE: min = -1.5, max = 50.0
Selected filters: None

Monoisotopic Mass, Even Electron Ions
11 formula(e) evaluated with 5 results within limits (all results (up to 1000) for each mass)
Elements Used:
C: 34-38 H: 40-50 N: 0-1 O: 12-16 Na: 0-1

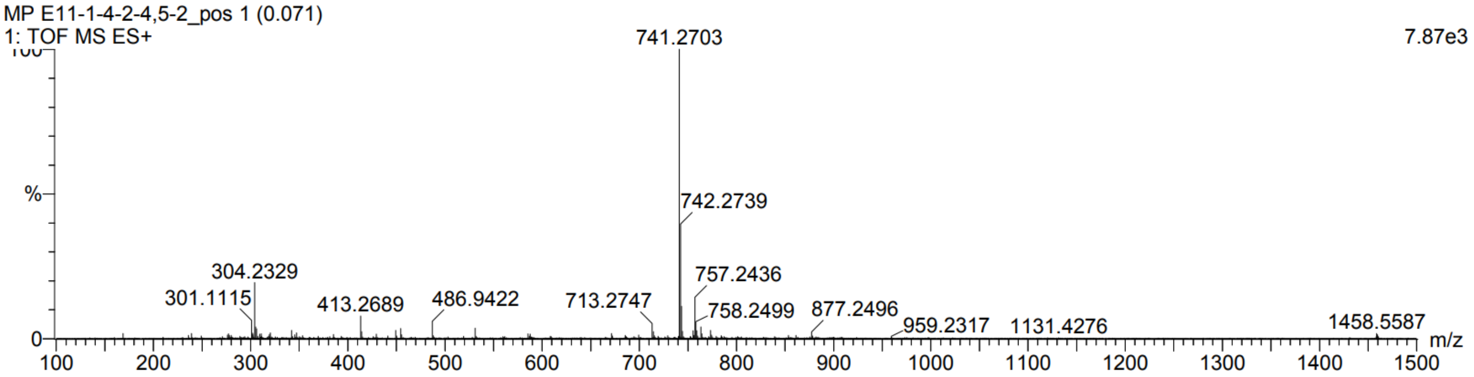

Minimum:				-1.5		
Maximum:		5.0	100.0	50.0		
Mass	Calc. Mass	mDa	PPM	DBE	i-FIT	Formula
741.2703	741.2734	-3.1	-4.2	13.5	2.8	C36 H46 O15 Na
	741.2758	-5.5	-7.4	16.5	14.6	C38 H45 O15
	741.2395	30.8	41.6	17.5	64.6	C37 H41 O16
	741.2371	33.2	44.8	14.5	67.2	C35 H42 O16 Na
	741.3098	-39.5	-53.3	12.5	98.4	C37 H50 O14 Na

Figure S18. HRESIMS of compound **3**.

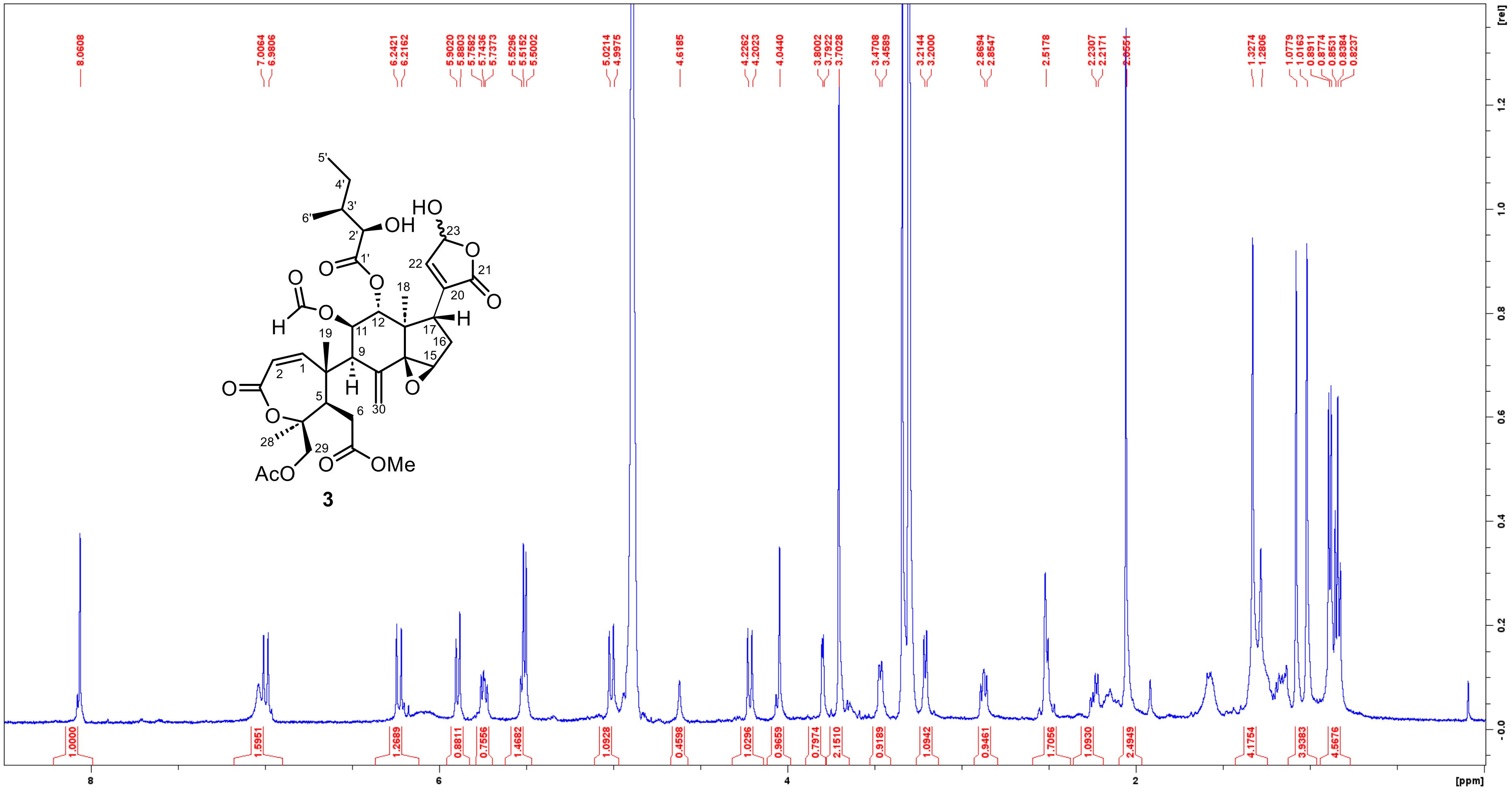

Figure S19. ^{1}H-NMR spectrum of compound **3** in CD_3OD.

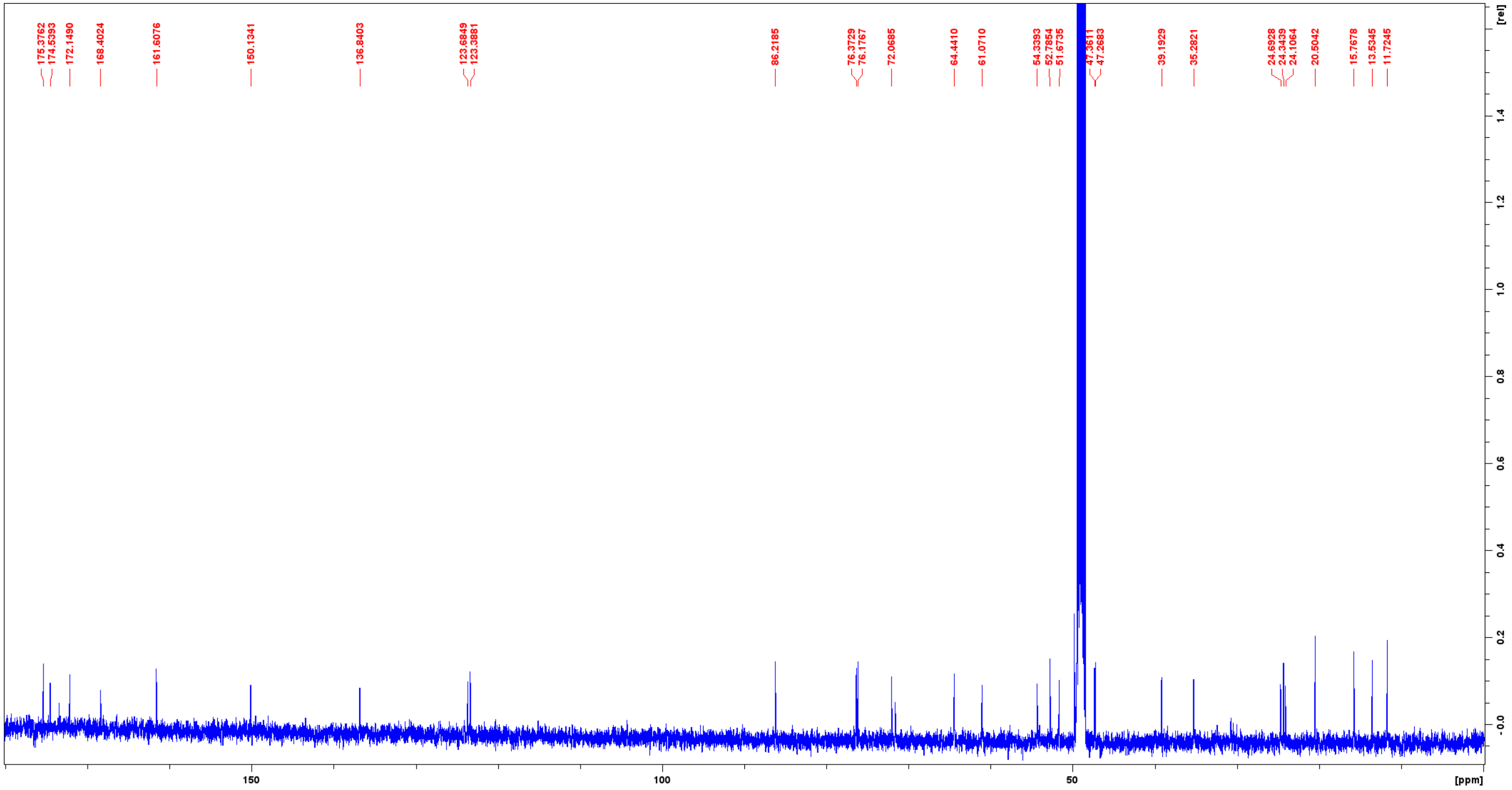

Figure S20. ^{13}C spectrum of compound **3** in CD_3OD.

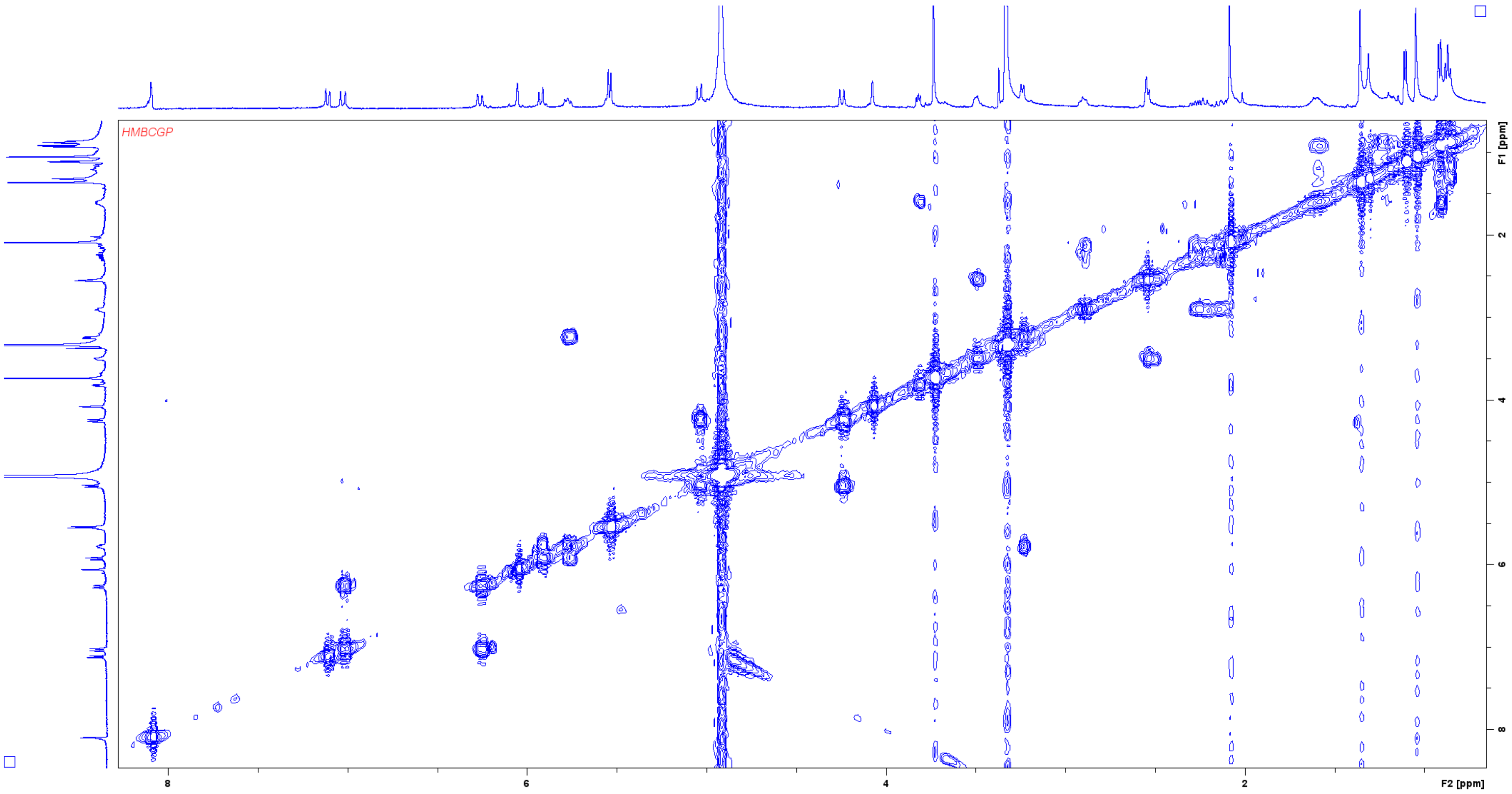

Figure S21. ^{1}H-^{1}H COSY spectrum of compound **3** in CD_3OD.

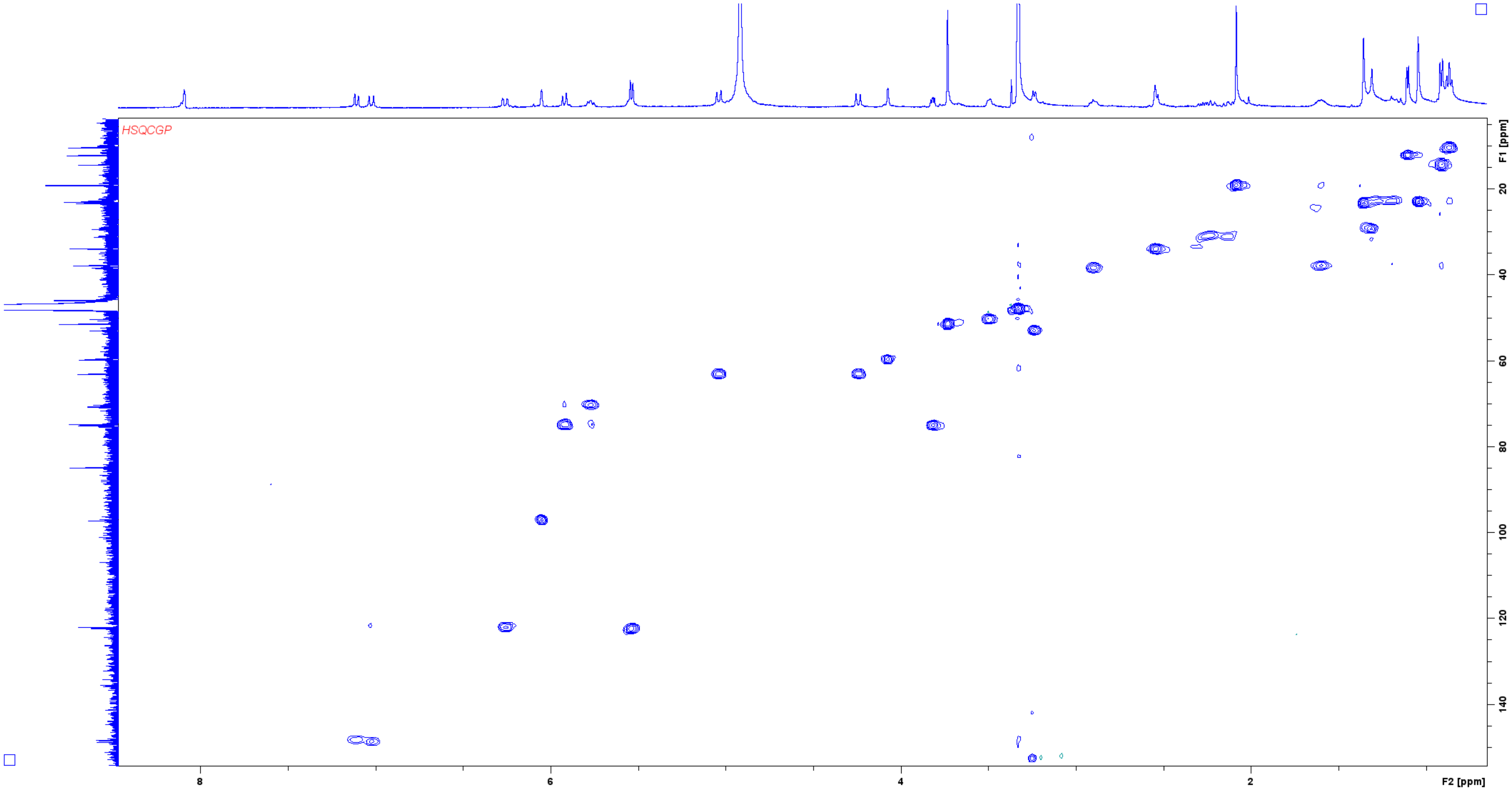

Figure S22. HSQC spectrum of compound **3** in CD_3OD.

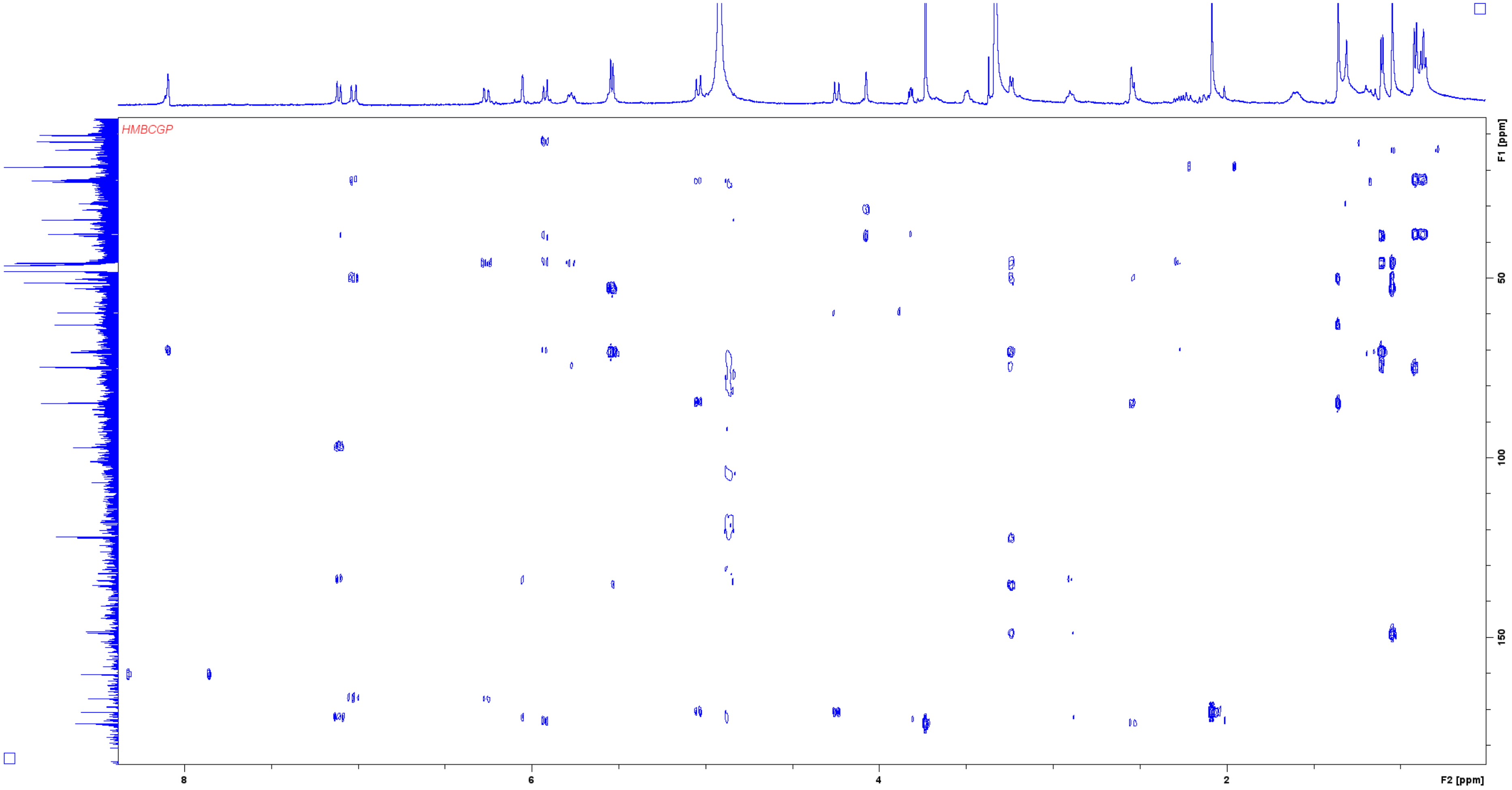

Figure S23. HMBC spectrum of compound **3** in CD_3OD.

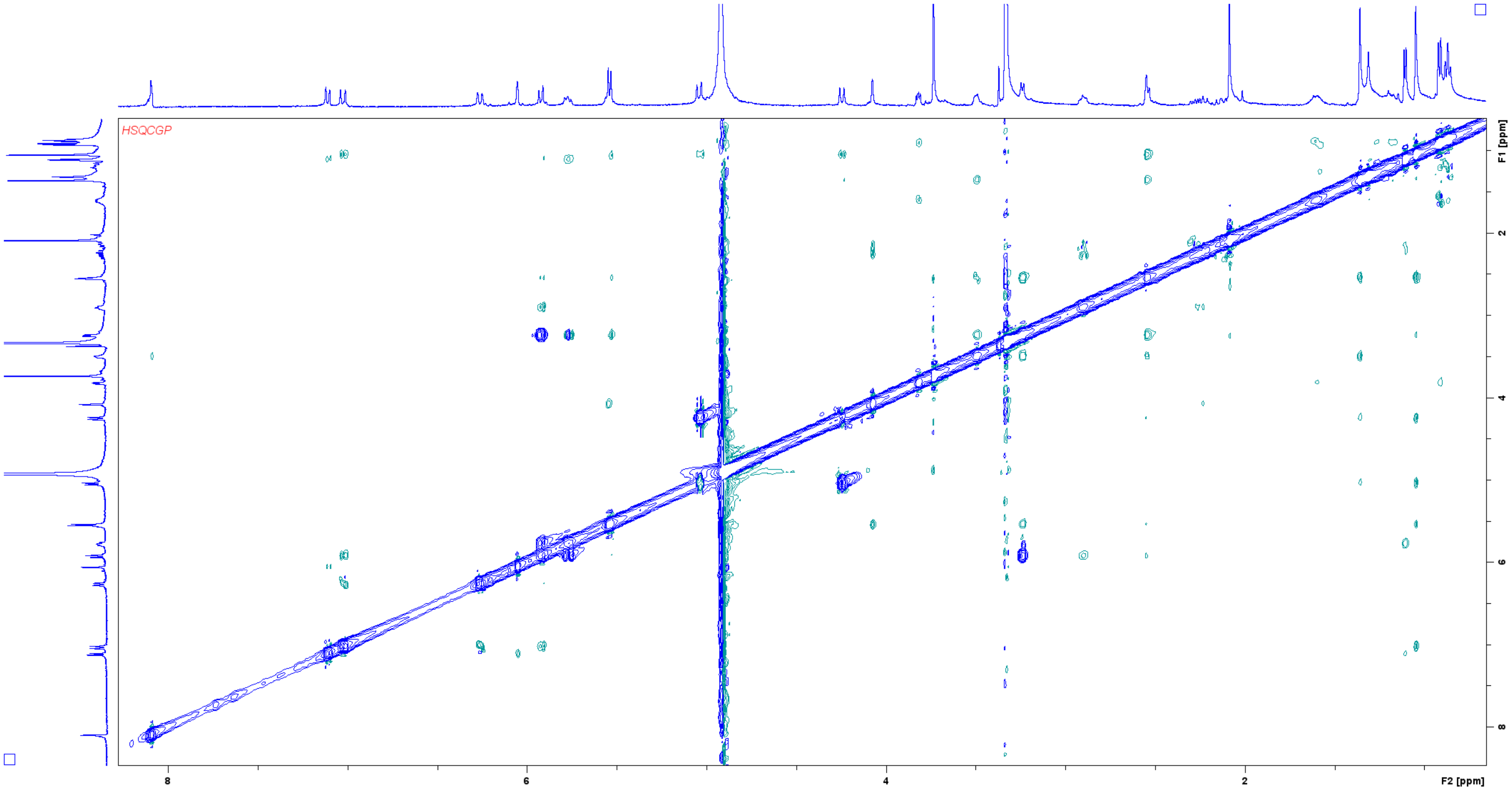

Figure S24. ROESY spectrum of compound **3** in CD_3OD**.**

Elemental Composition Report

Single Mass Analysis

Tolerance = 50.0 PPM / DBE: min = -1.5, max = 50.0
Selected filters: None

Monoisotopic Mass, Even Electron Ions
14 formula(e) evaluated with 7 results within limits (all results (up to 1000) for each mass)
Elements Used:
C: 32-38 H: 40-52 O: 11-17 Na: 1-1 K: 0-2

MP E11-1-4-2-4,5-3-POS 12 (0.541)
1: TOF MS ES+

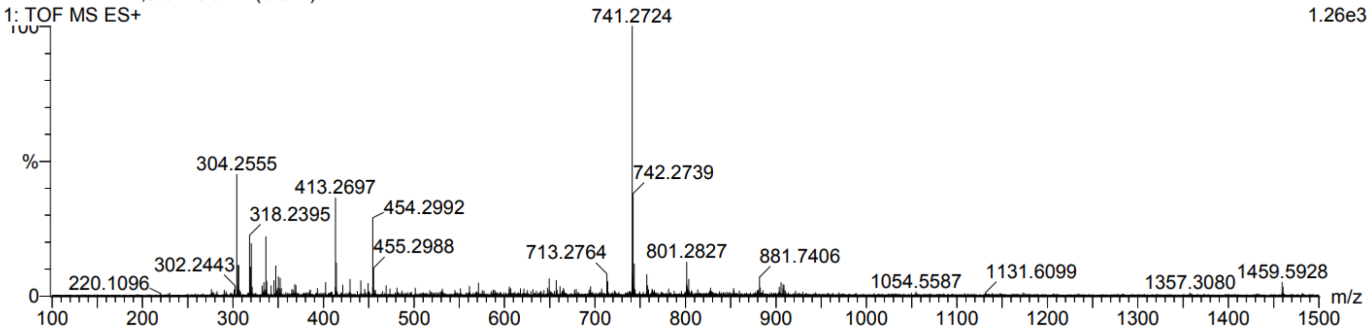

Minimum:				-1.5		
Maximum:		10.0	50.0	50.0		

Mass	Calc. Mass	mDa	PPM	DBE	i-FIT	Formula
741.2724	741.2734	-1.0	-1.3	13.5	1.8	C36 H46 O15 Na
	741.2946	-22.2	-29.9	8.5	6.2	C33 H50 O17 Na
	741.2371	35.3	47.6	14.5	12.4	C35 H42 O16 Na
	741.2864	-14.0	-18.9	9.5	16.4	C35 H51 O13 Na K
	741.2501	22.3	30.1	10.5	17.8	C34 H47 O14 Na K
	741.2653	7.1	9.6	14.5	19.6	C38 H47 O11 Na K
	741.2631	9.3	12.5	6.5	57.2	C33 H52 O12 Na K2

Figure S25. HRESIMS of compound **4**.

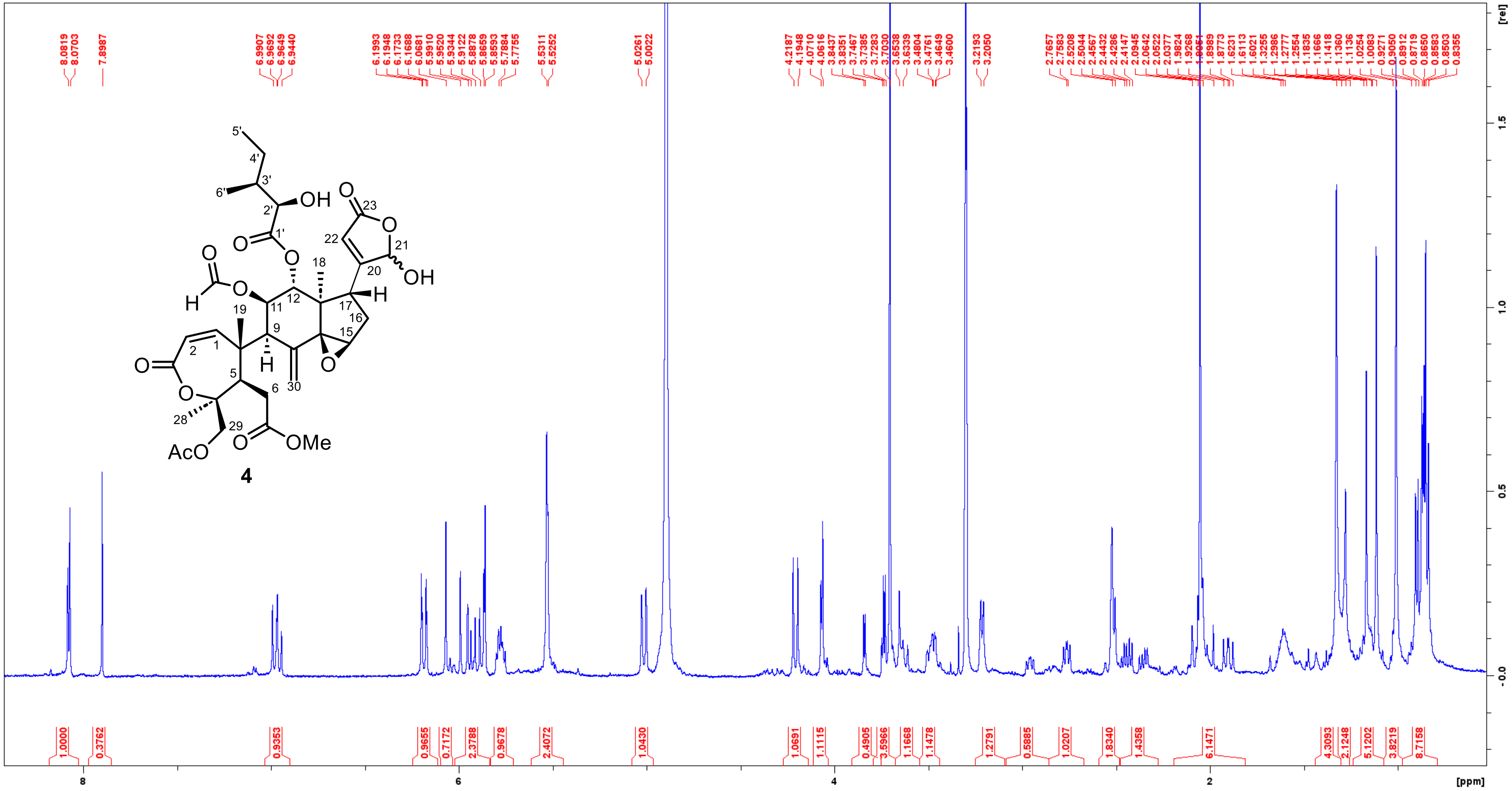

Figure S26. ^{1}H-NMR spectrum of compound **4** in CD_3OD.

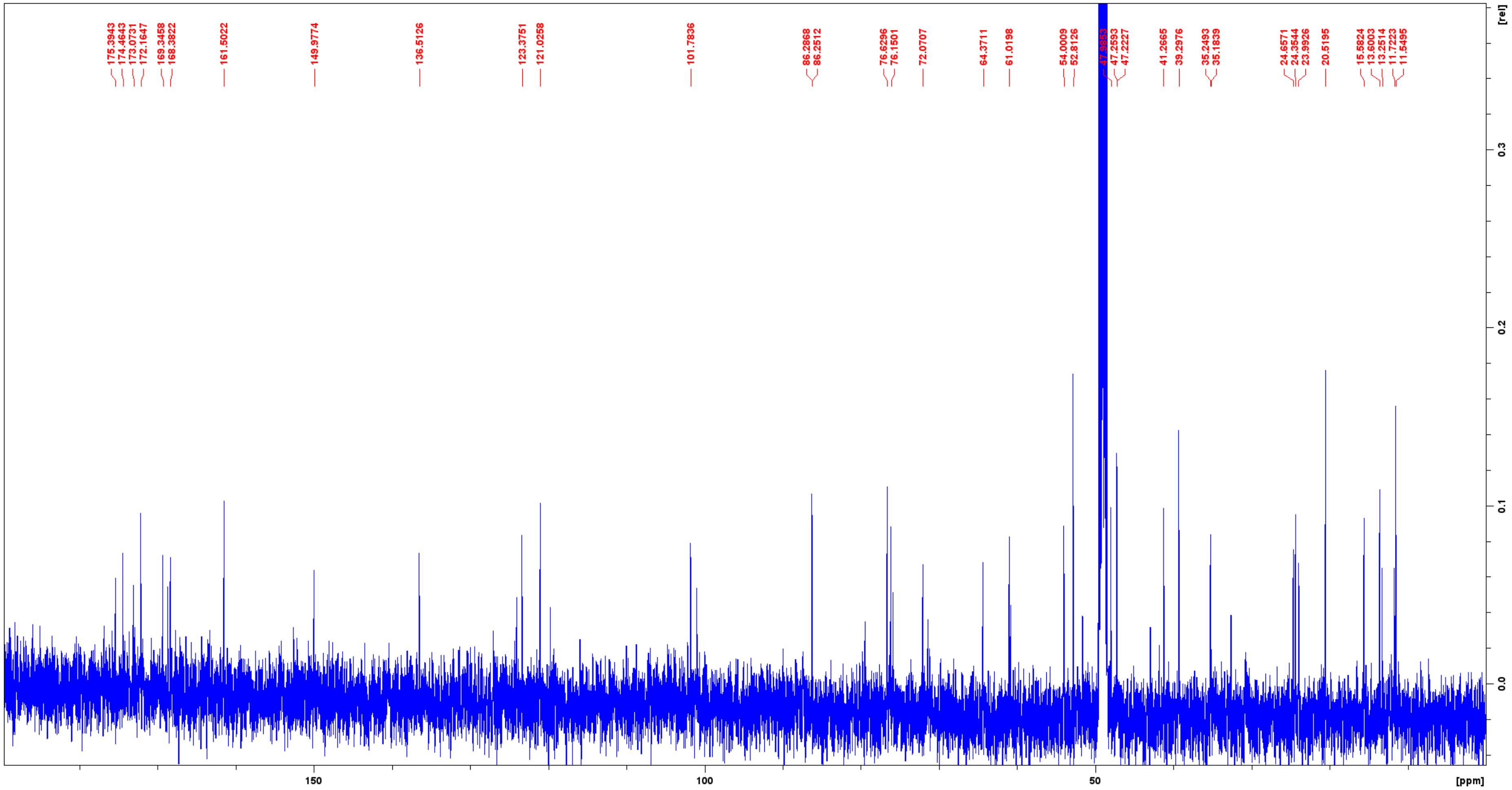

Figure S27. ^{13}C spectrum of compound **4** in CD_3OD.

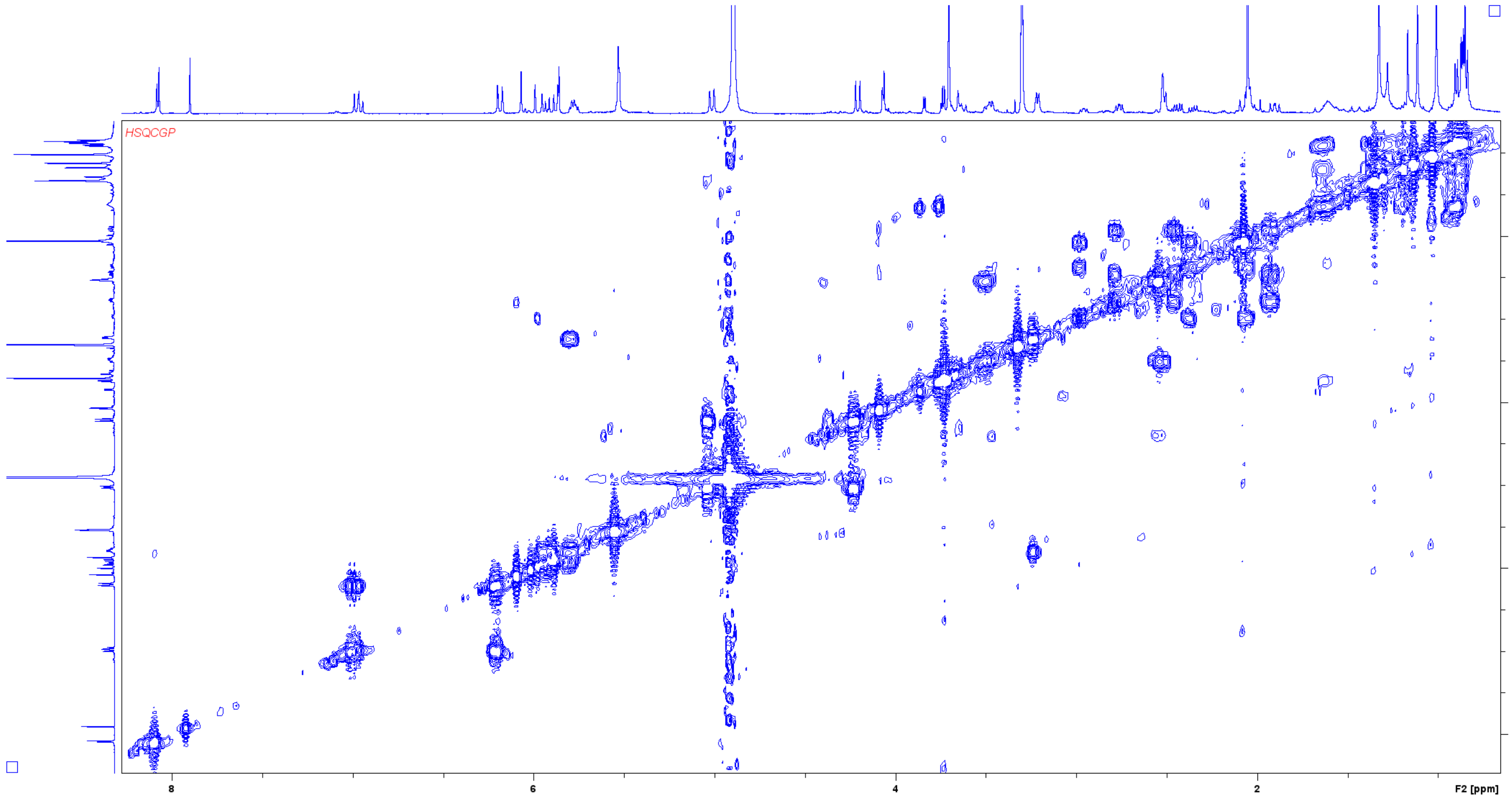

Figure S28. ^{1}H-^{1}H COSY spectrum of compound **4** in CD_3OD.

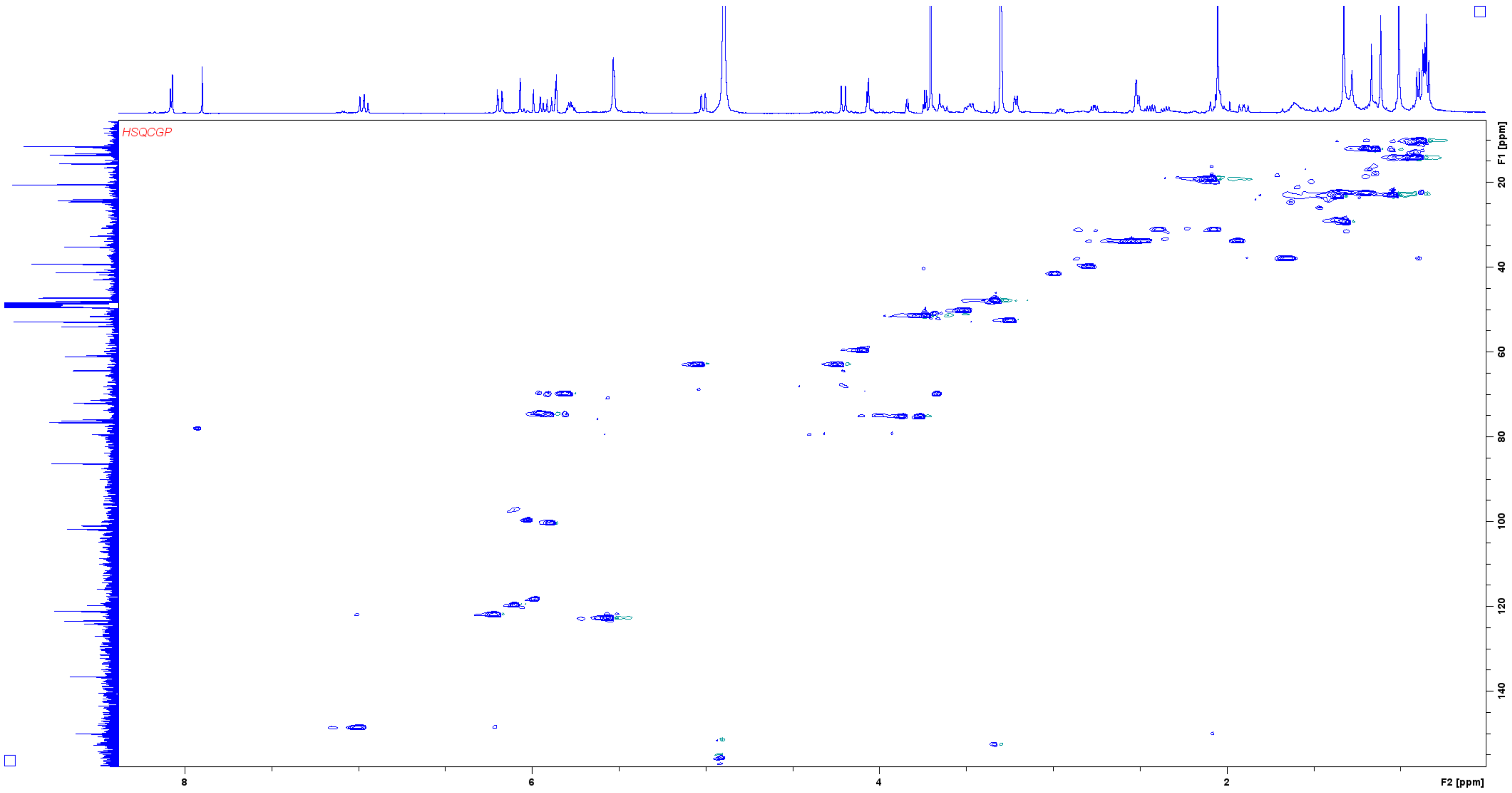

Figure S29. HSQC spectrum of compound **4** in CD_3OD.

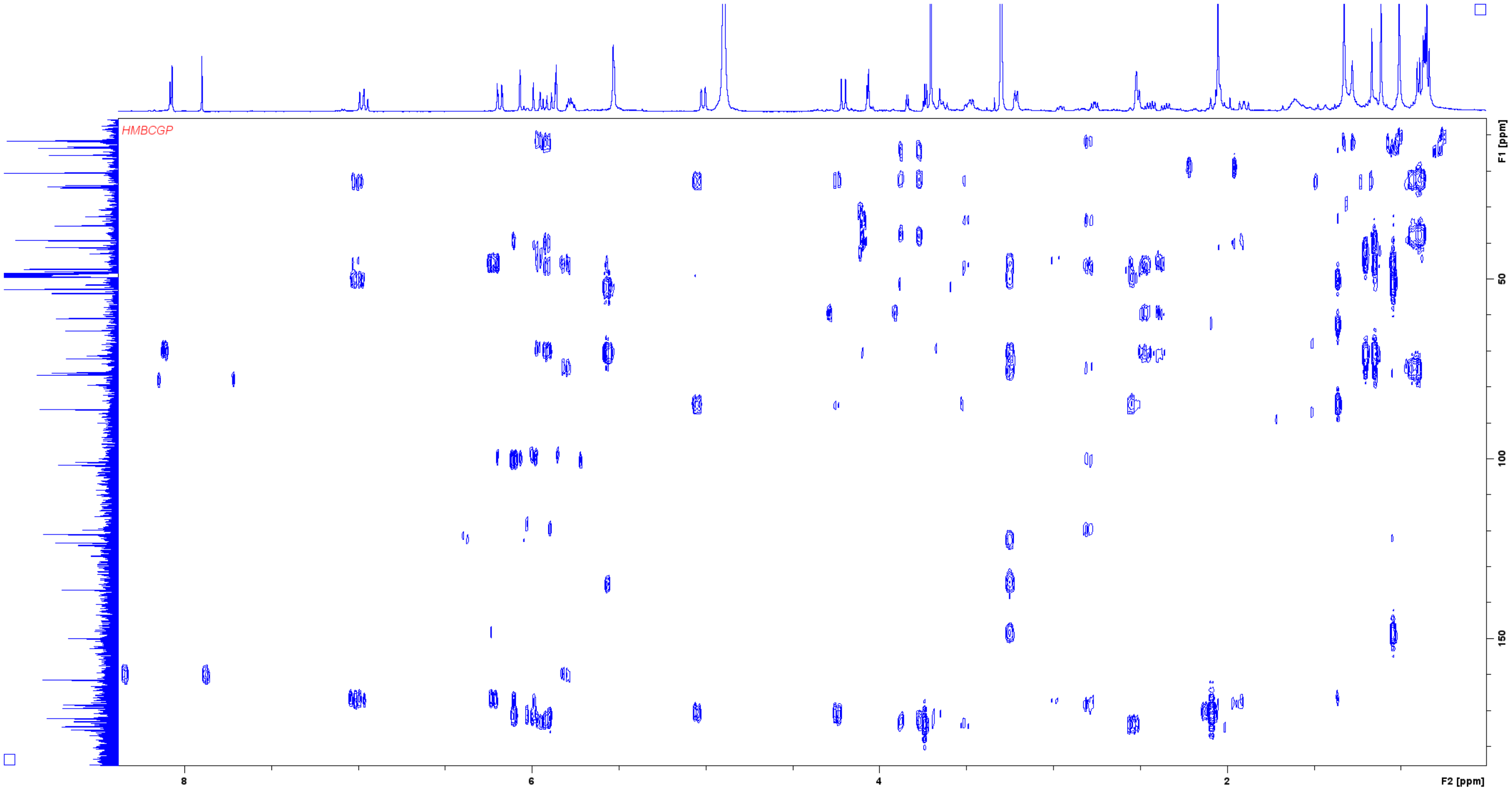

Figure S30. HMBC spectrum of compound **4** in CD_3OD.

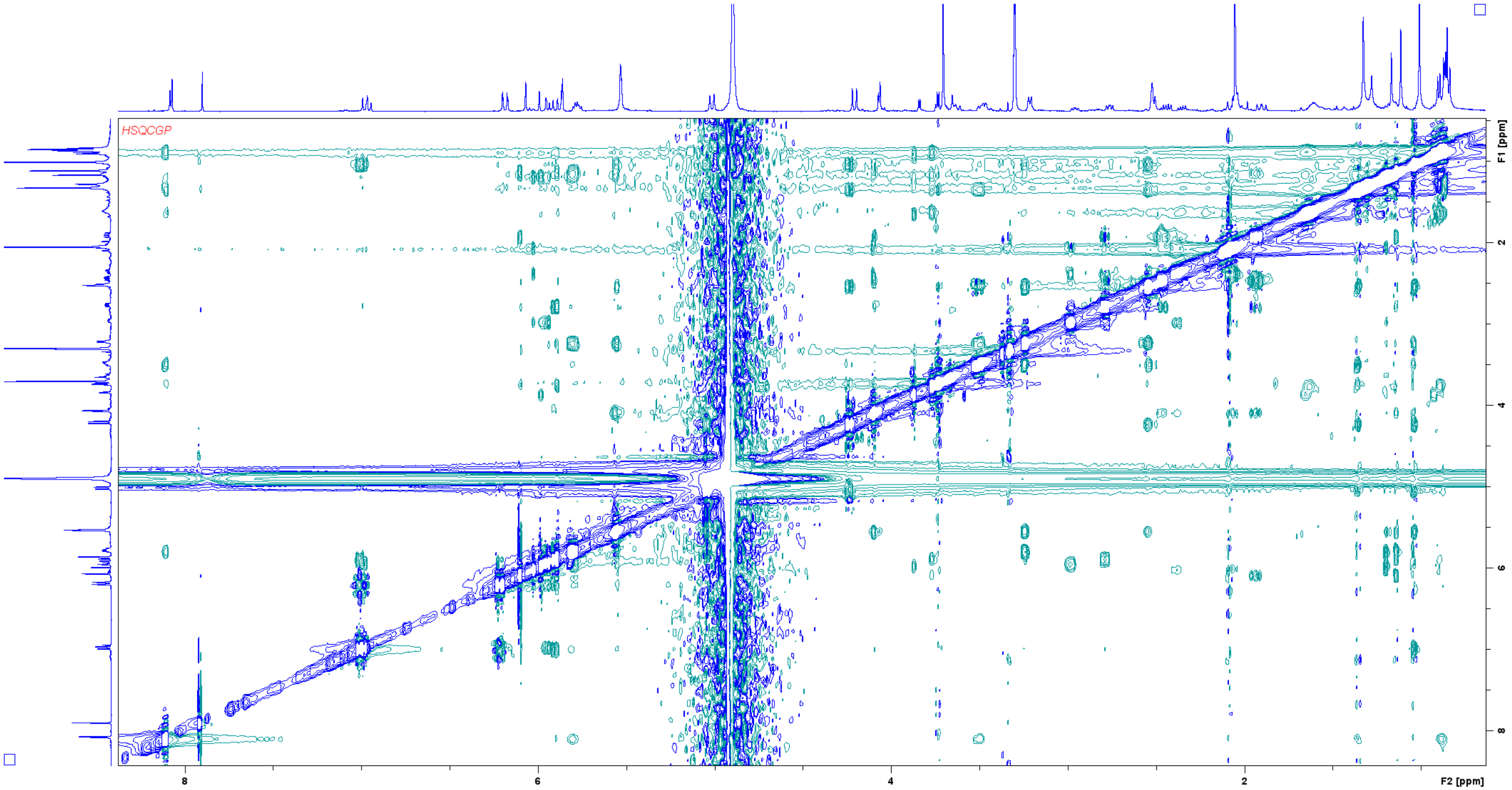

Figure S31. ROESY spectrum of compound **4** in CD_3OD.
